# Supplementary material for: Kinetic Isotope Effect in the Unfolding of a Protein Secondary Structure: Calculations for Beta-Sheet Polyglycine Dimers as a Model
Source: Biomolecules. 2025 Jan 9;15(1):92. doi: 10.3390/biom15010092 (PMC11764423; doi:10.3390/biom15010092)
Supplement: Supplementary file 1 [file biomolecules-15-00092-s001.zip › biomolecules-3360981-supplementary.pdf]

Supplementary Materials

S1. Estimations of the Anharmonicity Effect on the Strengthening of H-Bond for H/D Substitution

We have estimated influence of anharmonicity on the strengthening of H-bond $\delta_{light}^{heavy}(E_{H-bond})$ for H/D substitution with the approximation of quasidiatomic H-bond with Morse potential. As a model system we have used H-bond in water dimer. So-called empirical general hydrogen bond (GHB) potential combined from electrostatic and Lennard-Jones 6-12 contributions were built by McGuire et al. [1]. We have fitted this potential with Morse curve $U(R) = D_e \cdot (1 - e^{-\beta\cdot(R-R_e)})^2$ with parameters $D_e = 4.95$ kcal/mol, $R_e = 2.02$ Å and $\beta = 1.45$ Å^{-1} (see Figure S1). These parameters with the equation for zero-point energy of Morse oscillator from book of Herzberg [2] allow us to find the reduction of ZPE value due to anharmonicity of hydrogen bond by about 7.5 % for H-bond and about 5 % for D-bond. This corresponds to reduction of $\delta_{light}^{heavy}(E_{H-bond})$ value by about 10 % as compared with the number given in Table 1 of manuscript, which is calculated for H/D substitution within harmonic approximation.

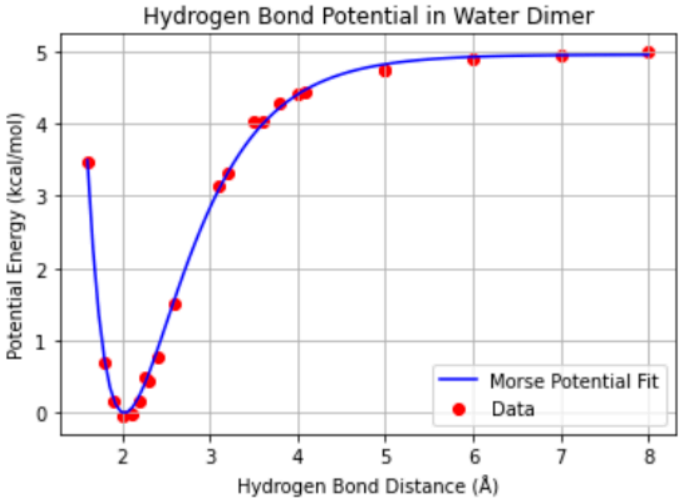


```
Fitting parameters (D_e, r_e, alpha): [4.95174459 2.02156226 1.44659873]
```

Figure S1. Fitting of the potential for H-bond in H_2O dimer from ref. [1] with the Morse curve $U(R) = D_e \cdot (1 - e^{-\beta\cdot(R-R_e)})^2$.

S2. The Influence on the Calculated Isotope Effect of Taking into Account the Dispersion Correction

In Figure S2 the change in the enthalpy ΔH_0^0 of polyglycine dimers dissociation upon H/D substitution (light/heavy) in the interchain H-bonds is presented which has been calculated with B3LYP-D3 approach. The slope value corresponds to a change of ΔH_0^0 per one link, which is designated in manuscript as δ(ZPE) per one link.

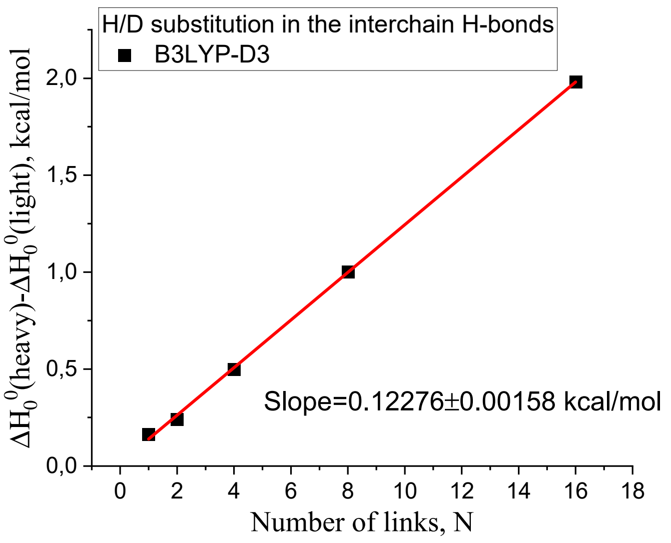

Figure S2. B3LYP-D3 **c**alculated change in the enthalpy ΔH_0^0 of polyglycine dimers dissociation upon isotopic substitution (light/heavy)

In Figure S3 the KIE values calculated with B3LYP and B3LYP-D3 approaches are presented. Presented data of B3LYP calculations show that H/D substitution in 1 link, containing 2 interchain H-bond, provides KIE value equal to 1.123. Calculations within B3LYP-D3 approach change this value by a factor of only 1.012. Therefore for longest dimer consisting of 16 links, the change from B3LYP to B3LYP-D3 approach results in the increase of the calculated KIE value by about 20 %.

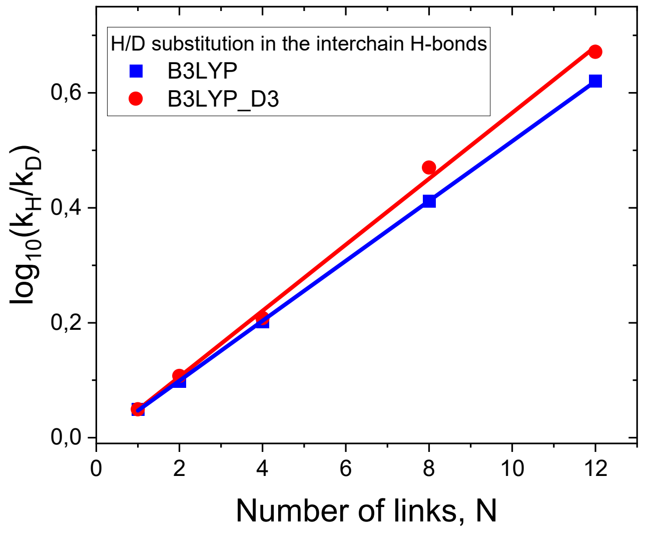

Figure S3. The KIE values calculated with B3LYP and B3LYP-D3 approaches.

S3. Raw Quantum Chemical Data: Optimized Geometries, Electronic Energies, and Thermal Corrections to Thermodynamic Potentials

B3LYP/6-31G(d) geometries, zero-point vibrational energies and thermal corrections to thermodynamic potentials

Monomer 1

B3LYP

Zero-point correction= 0.158277 (Hartree/Particle)
Thermal correction to Energy= 0.169327
Thermal correction to Enthalpy= 0.170271
Thermal correction to Gibbs Free Energy= 0.119199
Sum of electronic and zero-point Energies= -456.377895
Sum of electronic and thermal Energies= -456.366845
Sum of electronic and thermal Enthalpies= -456.365901
Sum of electronic and thermal Free Energies= -456.416974
E(RB3LYP) = -456.536172290

```
C 3.31380000 -1.20408500 0.00000000
C 2.34563100 -0.03053500 0.00000000
H 3.95814600 -1.13055900 0.88156600
H 2.82003600 -2.18075900 0.00000000
H 3.95814600 -1.13055900 -0.88156600
C 0.00000000 0.65527000 0.00000000
H 0.09486100 1.30840800 0.87911200
H 0.09486100 1.30840800 -0.87911200
C -1.36715500 -0.03526400 0.00000000
C -3.80870700 0.35507900 0.00000000
H -3.79072400 -0.73525500 0.00000000
H -4.34082400 0.70550300 0.89185800
O 2.72658600 1.13625400 0.00000000
N 1.02440700 -0.35730400 0.00000000
H 0.68785300 -1.31290800 0.00000000
O -1.47117300 -1.25879500 0.00000000
N -2.42955100 0.81327600 0.00000000
H -2.25024700 1.80794500 0.00000000
H -4.34082400 0.70550300 -0.89185800
```

B3LYP D3

Zero-point correction= 0.158252 (Hartree/Particle)
Thermal correction to Energy= 0.169343
Thermal correction to Enthalpy= 0.170287
Thermal correction to Gibbs Free Energy= 0.118785
Sum of electronic and zero-point Energies= -456.390689
Sum of electronic and thermal Energies= -456.379598
Sum of electronic and thermal Enthalpies= -456.378654
Sum of electronic and thermal Free Energies= -456.430155
E(RB3LYP) = -456.548941

```
C 3.32833400 -1.18827400 0.00000000
C 2.34459200 -0.02773900 0.00000000
H 3.97093500 -1.10244900 0.88164400
H 2.85086500 -2.17321800 0.00000000
H 3.97093500 -1.10244900 -0.88164400
C 0.00000000 0.64183200 0.00000000
H 0.09607900 1.29513900 0.87882700
H 0.09607900 1.29513900 -0.87882700
C -1.36919700 -0.04460000 0.00000000
C -3.80888600 0.36533500 0.00000000
H -3.80371200 -0.72534400 0.00000000
H -4.33701600 0.72113200 0.89214200
O 2.71168600 1.14343400 0.00000000
N 1.02706700 -0.36796800 0.00000000
H 0.69993500 -1.32654300 0.00000000
O -1.48014800 -1.26752300 0.00000000
N -2.42556200 0.81166500 0.00000000
H -2.23898800 1.80497200 0.00000000
```

H -4.33701600 0.72113200 -0.89214200

Dimer 1

B3LYP

Zero-point correction= 0.321359 (Hartree/Particle)
Thermal correction to Energy= 0.343701
Thermal correction to Enthalpy= 0.344645
Thermal correction to Gibbs Free Energy= 0.265898
Sum of electronic and zero-point Energies= -912.786618
Sum of electronic and thermal Energies= -912.764277
Sum of electronic and thermal Enthalpies= -912.763333
Sum of electronic and thermal Free Energies= -912.842079
E(RB3LYP)= -913.107977

```
C 2.77536200 -3.02378000 0.65472000
C 2.85729100 -1.70083000 -0.08389000
H 3.23421200 -3.80116000 0.03491000
H 3.36205200 -2.95494000 1.57590000
H 1.74943200 -3.31585100 0.89436000
C 1.60035000 0.07312900 -1.21729000
H 2.32962000 -0.00830000 -2.03129000
H 0.59772000 0.14030900 -1.64234000
C 1.86103900 1.36683900 -0.42551000
C 3.59650800 2.66284000 0.74259000
H 2.79565800 3.40208000 0.78242000
H 3.83092900 2.33587000 1.76339000
O 3.94425100 -1.18832900 -0.38261000
N 1.66983100 -1.12728100 -0.39638000
H 0.78562100 -1.57499100 -0.13532000
O 0.94962900 2.16062900 -0.14605000
N 3.15001900 1.55154000 -0.07927000
H 3.77449000 0.76188000 -0.25475000
H 4.49152800 3.11914100 0.30744000
C -3.59714900 -2.66254400 -0.74254000
H -4.48322800 -3.12862400 -0.29933000
H -3.84629900 -2.33229400 -1.75871000
H -2.79074800 -3.39492300 -0.79516000
C -1.59972000 -0.07321300 1.21665000
H -2.32857000 0.00813700 2.03106000
H -0.59687000 -0.14025200 1.64120000
C -2.77593200 3.02396700 -0.65414000
H -1.75014200 3.31614700 -0.89425000
H -3.36310200 2.95533600 -1.57502000
H -3.23447200 3.80117700 -0.03388000
N -3.14975900 -1.55129300 0.07889000
H -3.77412000 -0.76174400 0.25522000
C -1.86070900 -1.36707300 0.42520000
O -0.94945900 -2.16112200 0.14598000
N -1.66976100 1.12716700 0.39575000
H -0.78569100 1.57509800 0.13461000
O -3.94419100 1.18830600 0.38350000
C -2.85740100 1.70084700 0.08421000
```

B3LYP-D3

Zero-point correction= 0.321716 (Hartree/Particle)
Thermal correction to Energy= 0.343956
Thermal correction to Enthalpy= 0.344900
Thermal correction to Gibbs Free Energy= 0.265718
Sum of electronic and zero-point Energies= -912.824268
Sum of electronic and thermal Energies= -912.802028
Sum of electronic and thermal Enthalpies= -912.801084
Sum of electronic and thermal Free Energies= -912.880266
E(RB3LYP)= -913.145984

```
C 2.51564700 -3.10044700 0.64383900
C 2.68390900 -1.78414400 -0.09186400
H 2.92700300 -3.90265800 0.02255100
H 3.10510200 -3.06761800 1.56515900
H 1.47222200 -3.32643400 0.88043700
C 1.53460500 0.06594000 -1.22009000
H 2.24060100 -0.06103100 -2.04920000
```

```
H 0.53006000 0.20666000 -1.62230900
C 1.89974800 1.32995500 -0.42339500
C 3.71915000 2.49034500 0.74625900
H 3.06228100 3.35720500 0.65924700
H 3.75894400 2.18507700 1.79993100
O 3.80202000 -1.34152300 -0.38737000
N 1.53453400 -1.13591700 -0.39927800
H 0.62922600 -1.55947800 -0.16903700
O 1.04837600 2.17934000 -0.11563800
N 3.20187300 1.42683500 -0.09777600
H 3.76824100 0.59469300 -0.27468100
H 4.72721100 2.76119100 0.41965400
C -3.71891900 -2.49040800 -0.74630300
H -4.72747400 -2.76053500 -0.42065300
H -3.75741600 -2.18551200 -1.80013300
H -3.06265400 -3.35763000 -0.65830800
C -1.53491200 -0.06584500 1.22042100
H -2.24113400 0.06120900 2.04932200
H -0.53048000 -0.20657500 1.62292300
C -2.51537300 3.10026200 -0.64430300
H -1.47186200 3.32623100 -0.88053900
H -3.10445200 3.06718400 -1.56585700
H -2.92703000 3.90259400 -0.02337500
N -3.20195400 -1.42689600 0.09792800
H -3.76837300 -0.59473800 0.27458800
C -1.89986800 -1.32988200 0.42367600
O -1.04840300 -2.17918600 0.11597100
N -1.53458400 1.13595400 0.39952700
H -0.62922600 1.55942600 0.16932300
O -3.80206700 1.34156600 0.38691000
C -2.68386900 1.78411300 0.09162400
```

Monomer 2

B3LYP

Zero-point correction= 0.269606 (Hartree/Particle)

Thermal correction to Energy= 0.289701

Thermal correction to Enthalpy= 0.290645

Thermal correction to Gibbs Free Energy= 0.214305

Sum of electronic and zero-point Energies= -872.291652

Sum of electronic and thermal Energies= -872.271556

Sum of electronic and thermal Enthalpies= -872.270612

Sum of electronic and thermal Free Energies= -872.346953

E(RB3LYP)= -872.561257

```
C -6.96679600 -1.44781600 0.00000000
C -5.56124900 -2.03172800 0.00000000
H -7.50181600 -1.81470300 0.88148000
H -6.98816600 -0.35361000 0.00000000
H -7.50181600 -1.81470300 -0.88148000
C -3.16567800 -1.53080400 0.00000000
H -2.93751300 -2.14979400 0.87882000
H -2.93751300 -2.14979400 -0.87882000
C -2.29377400 -0.27232300 0.00000000
C 0.00000000 0.58100900 0.00000000
H -0.13085100 1.22496000 -0.88011000
O -5.35345400 -3.24139300 0.00000000
N -4.54823700 -1.12361700 0.00000000
H -4.70122700 -0.12191000 0.00000000
O -2.78141100 0.85753000 0.00000000
N -0.96058500 -0.49625200 0.00000000
H -0.55793100 -1.42849400 0.00000000
H -0.13085100 1.22496000 0.88011000
C 1.40149500 -0.03748500 0.00000000
O 1.56964200 -1.25638800 0.00000000
N 2.42246200 0.84654900 0.00000000
C 3.80215400 0.42290000 0.00000000
H 2.28527200 1.85303900 0.00000000
H 4.02117000 -0.19581200 0.88088000
H 4.02117000 -0.19581200 -0.88088000
C 4.68169000 1.67938300 0.00000000
O 4.18567400 2.80266400 0.00000000
N 6.01720200 1.43796500 0.00000000
C 7.01387100 2.49738300 0.00000000
H 6.33351300 0.47788400 0.00000000
H 6.48145300 3.44886700 0.00000000
```

H 7.64766300 2.43999200 0.89198000
H 7.64766300 2.43999200 -0.89198000

B3LYP-D3

Zero-point correction= 0.269544 (Hartree/Particle)
Thermal correction to Energy= 0.289722
Thermal correction to Enthalpy= 0.290666
Thermal correction to Gibbs Free Energy= 0.213620
Sum of electronic and zero-point Energies= -872.318403
Sum of electronic and thermal Energies= -872.298225
Sum of electronic and thermal Enthalpies= -872.297281
Sum of electronic and thermal Free Energies= -872.374327
E(RB3LYP)= -872.587947

C -6.97888000 -1.43884200 0.00000000
C -5.56859200 -2.01109300 0.00000000
H -7.50840600 -1.81290800 0.88166500
H -7.01279200 -0.34474800 0.00000000
H -7.50840600 -1.81290800 -0.88166500
C -3.17889000 -1.50934300 0.00000000
H -2.95521300 -2.13049900 0.87840300
H -2.95521300 -2.13049900 -0.87840300
C -2.29593200 -0.25932100 0.00000000
C 0.00000000 0.57278000 0.00000000
H -0.13075000 1.21718900 -0.87975100
O -5.35301000 -3.21939300 0.00000000
N -4.56024900 -1.09813800 0.00000000
H -4.71762600 -0.09749500 0.00000000
O -2.76796700 0.87710900 0.00000000
N -0.96577600 -0.49956900 0.00000000
H -0.57619400 -1.43680400 0.00000000
H -0.13075000 1.21718900 0.87975100
C 1.40180900 -0.04362300 0.00000000
O 1.57739400 -1.26143900 0.00000000
N 2.41845200 0.84511200 0.00000000
C 3.79695800 0.41837800 0.00000000
H 2.27476400 1.85016800 0.00000000
H 4.01152900 -0.20238500 0.88052700
H 4.01152900 -0.20238500 -0.88052700
C 4.68701000 1.66735200 0.00000000
O 4.20416200 2.79623700 0.00000000
N 6.01965000 1.40937800 0.00000000
C 7.03063500 2.45542700 0.00000000
H 6.32405700 0.44549200 0.00000000
H 6.51342700 3.41554200 0.00000000
H 7.66308400 2.38858800 0.89230600
H 7.66308400 2.38858800 -0.89230600

Dimer 2

B3LYP

Zero-point correction= 0.547781 (Hartree/Particle)
Thermal correction to Energy= 0.586585
Thermal correction to Enthalpy= 0.587530
Thermal correction to Gibbs Free Energy= 0.469902
Sum of electronic and zero-point Energies= -1744.624090
Sum of electronic and thermal Energies= -1744.585286
Sum of electronic and thermal Enthalpies= -1744.584342
Sum of electronic and thermal Free Energies= -1744.701970
E(RB3LYP)= -1745.171872

C 6.05110000 -2.95490000 -0.74572000
C 4.74642000 -2.89799000 0.02654000
H 6.80698000 -3.44396000 -0.12287000
H 5.91208000 -3.57594000 -1.63598000
H 6.41807000 -1.96913000 -1.04466000
C 3.04702000 -1.48220000 1.06267000
H 3.07987000 -2.15162000 1.92848000
H 3.01593000 -0.44677000 1.40279000
C 1.77904000 -1.74518000 0.24304000
C 0.31184000 -3.45476000 -0.71664000

```
H 0.45661000 -4.49543000 -1.00717000
O 4.17662000 -3.92536000 0.41568000
N 4.25387000 -1.66008000 0.27396000
H 4.67494000 -0.80612000 -0.09239000
O 1.12936000 -0.83152000 -0.27954000
N 1.42876000 -3.04611000 0.12719000
H 2.13193000 -3.72921000 0.40830000
H 0.29020000 -2.82474000 -1.61375000
C 5.49042000 3.67244000 0.78598000
H 6.01725000 4.49644000 0.29214000
H 5.24701000 3.97815000 1.81084000
H 6.14109000 2.79781000 0.81887000
C 2.68135000 1.90678000 -1.10327000
H 2.65789000 2.60487000 -1.94895000
H 2.62337000 0.88464000 -1.47369000
C -0.31184000 3.45476000 0.71664000
H -0.45661000 4.49543000 1.00717000
N 4.29231000 3.31716000 0.04716000
H 3.55232000 4.00660000 -0.09415000
C 4.02870000 2.06274000 -0.37218000
O 4.80509000 1.10985000 -0.21811000
N 1.50709000 2.13370000 -0.26660000
H 0.94538000 1.31534000 -0.02822000
O 1.66892000 4.40708000 -0.28868000
C 1.04774000 3.37743000 0.00150000
C -1.04774000 -3.37743000 -0.00150000
O -1.66892000 -4.40708000 0.28868000
N -1.50709000 -2.13370000 0.26660000
C -2.68135000 -1.90678000 1.10327000
H -0.94538000 -1.31534000 0.02822000
H -2.65789000 -2.60487000 1.94895000
H -2.62337000 -0.88464000 1.47369000
C -4.02870000 -2.06274000 0.37218000
O -4.80509000 -1.10985000 0.21811000
N -4.29231000 -3.31716000 -0.04716000
C -5.49042000 -3.67244000 -0.78598000
H -3.55232000 -4.00660000 0.09415000
H -5.24701000 -3.97815000 -1.81084000
H -6.01725000 -4.49644000 -0.29214000
H -6.14109000 -2.79781000 -0.81887000
H -0.29020000 2.82474000 1.61375000
N -1.42876000 3.04611000 -0.12719000
H -2.13193000 3.72921000 -0.40830000
C -1.77904000 1.74518000 -0.24304000
O -1.12936000 0.83152000 0.27954000
C -3.04702000 1.48220000 -1.06267000
H -3.01593000 0.44677000 -1.40279000
H -3.07987000 2.15162000 -1.92848000
C -4.74642000 2.89799000 -0.02654000
O -4.17662000 3.92536000 -0.41568000
C -6.05110000 2.95490000 0.74572000
H -5.91208000 3.57594000 1.63598000
H -6.80698000 3.44396000 0.12287000
H -6.41807000 1.96913000 1.04466000
N -4.25387000 1.66008000 -0.27396000
H -4.67494000 0.80612000 0.09239000
```

B3LYP-D3

Zero-point correction= 0.548018 (Hartree/Particle)
Thermal correction to Energy= 0.586875
Thermal correction to Enthalpy= 0.587819
Thermal correction to Gibbs Free Energy= 0.467851
Sum of electronic and zero-point Energies= -1744.704552
Sum of electronic and thermal Energies= -1744.665695
Sum of electronic and thermal Enthalpies= -1744.664751
Sum of electronic and thermal Free Energies= -1744.784719
E(RB3LYP)= -1745.252570

```
C 6.05110000 -2.95490000 -0.74572000
C 4.74642000 -2.89799000 0.02654000
H 6.80698000 -3.44396000 -0.12287000
H 5.91208000 -3.57594000 -1.63598000
H 6.41807000 -1.96913000 -1.04466000
C 3.04702000 -1.48220000 1.06267000
H 3.07987000 -2.15162000 1.92848000
H 3.01593000 -0.44677000 1.40279000
C 1.77904000 -1.74518000 0.24304000
```

C 0.31184000 -3.45476000 -0.71664000
H 0.45661000 -4.49543000 -1.00717000
O 4.17662000 -3.92536000 0.41568000
N 4.25387000 -1.66008000 0.27396000
H 4.67494000 -0.80612000 -0.09239000
O 1.12936000 -0.83152000 -0.27954000
N 1.42876000 -3.04611000 0.12719000
H 2.13193000 -3.72921000 0.40830000
H 0.29020000 -2.82474000 -1.61375000
C 5.49042000 3.67244000 0.78598000
H 6.01725000 4.49644000 0.29214000
H 5.24701000 3.97815000 1.81084000
H 6.14109000 2.79781000 0.81887000
C 2.68135000 1.90678000 -1.10327000
H 2.65789000 2.60487000 -1.94895000
H 2.62337000 0.88464000 -1.47369000
C -0.31184000 3.45476000 0.71664000
H -0.45661000 4.49543000 1.00717000
N 4.29231000 3.31716000 0.04716000
H 3.55232000 4.00660000 -0.09415000
C 4.02870000 2.06274000 -0.37218000
O 4.80509000 1.10985000 -0.21811000
N 1.50709000 2.13370000 -0.26660000
H 0.94538000 1.31534000 -0.02822000
O 1.66892000 4.40708000 -0.28868000
C 1.04774000 3.37743000 0.00150000
C -1.04774000 -3.37743000 -0.00150000
O -1.66892000 -4.40708000 0.28868000
N -1.50709000 -2.13370000 0.26660000
C -2.68135000 -1.90678000 1.10327000
H -0.94538000 -1.31534000 0.02822000
H -2.65789000 -2.60487000 1.94895000
H -2.62337000 -0.88464000 1.47369000
C -4.02870000 -2.06274000 0.37218000
O -4.80509000 -1.10985000 0.21811000
N -4.29231000 -3.31716000 -0.04716000
C -5.49042000 -3.67244000 -0.78598000
H -3.55232000 -4.00660000 0.09415000
H -5.24701000 -3.97815000 -1.81084000
H -6.01725000 -4.49644000 -0.29214000
H -6.14109000 -2.79781000 -0.81887000
H -0.29020000 2.82474000 1.61375000
N -1.42876000 3.04611000 -0.12719000
H -2.13193000 3.72921000 -0.40830000
C -1.77904000 1.74518000 -0.24304000
O -1.12936000 0.83152000 0.27954000
C -3.04702000 1.48220000 -1.06267000
H -3.01593000 0.44677000 -1.40279000
H -3.07987000 2.15162000 -1.92848000
C -4.74642000 2.89799000 -0.02654000
O -4.17662000 3.92536000 -0.41568000
C -6.05110000 2.95490000 0.74572000
H -5.91208000 3.57594000 1.63598000
H -6.80698000 3.44396000 0.12287000
H -6.41807000 1.96913000 1.04466000
N -4.25387000 1.66008000 -0.27396000
H -4.67494000 0.80612000 0.09239000

Monomer 4

B3LYP

Zero-point correction= 0.492285 (Hartree/Particle)
Thermal correction to Energy= 0.530486
Thermal correction to Enthalpy= 0.531430
Thermal correction to Gibbs Free Energy= 0.405017
Sum of electronic and zero-point Energies= -1704.120573
Sum of electronic and thermal Energies= -1704.082373
Sum of electronic and thermal Enthalpies= -1704.081429
Sum of electronic and thermal Free Energies= -1704.207842
E(RB3LYP)= -1704.612859

C -13.90263600 -3.48587500 0.00000000
C -12.49417400 -4.06333400 0.00000000
H -14.43594600 -3.85521100 0.88151000
H -13.92912000 -2.39176600 0.00000000
H -14.43594600 -3.85521100 -0.88151000
C -10.10060600 -3.54809300 0.00000000

```
H -9.86807700 -4.16528900 0.87886000
H -9.86807700 -4.16528900 -0.87886000
C -9.23832400 -2.28244400 0.00000000
C -6.94966100 -1.41272900 0.00000000
H -7.08419100 -0.76989500 -0.88026000
O -12.28157800 -5.27226300 0.00000000
N -11.48607700 -3.15033200 0.00000000
H -11.64409100 -2.14930000 0.00000000
O -9.73667700 -1.15686100 0.00000000
N -7.90421300 -2.49565800 0.00000000
H -7.49312600 -3.42454200 0.00000000
H -7.08419100 -0.76989500 0.88026000
C -5.54455000 -2.02563600 0.00000000
O -5.37442700 -3.24454200 0.00000000
N -4.52938100 -1.13680700 0.00000000
C -3.14382300 -1.54450800 0.00000000
H -4.67352300 -0.13088000 0.00000000
H -2.91524400 -2.15939200 0.88058000
H -2.91524400 -2.15939200 -0.88058000
C -2.28512000 -0.27406700 0.00000000
O -2.79668500 0.84552900 0.00000000
N -0.95247700 -0.48026100 0.00000000
C 0.00000000 0.60582900 0.00000000
H -0.53478800 -1.40685700 0.00000000
H -0.13584400 1.24729300 -0.88072000
H -0.13584400 1.24729300 0.88072000
C 1.40469700 -0.00958900 0.00000000
C 3.80648100 0.46516900 0.00000000
H 4.03356100 -0.14985500 0.88077000
H 4.03356100 -0.14985500 -0.88077000
C 4.66629500 1.73496900 0.00000000
C 6.95074200 2.61503500 0.00000000
H 6.81449000 3.25641000 -0.88071000
O 1.56930900 -1.22947300 0.00000000
N 2.42144500 0.87592000 0.00000000
H 2.28042300 1.88250600 0.00000000
O 4.15502900 2.85451600 0.00000000
N 5.99886800 1.52827500 0.00000000
H 6.41591600 0.60150900 0.00000000
H 6.81449000 3.25641000 0.88071000
C 8.35543200 2.00105800 0.00000000
O 8.52266500 0.78163300 0.00000000
N 9.37277000 2.88677700 0.00000000
C 10.75523500 2.47024700 0.00000000
H 9.23327800 3.89337200 0.00000000
H 10.97747400 1.85340400 0.88119000
H 10.97747400 1.85340400 -0.88119000
C 11.62506600 3.73423300 0.00000000
O 11.11758000 4.85252900 0.00000000
N 12.96195200 3.50494800 0.00000000
C 13.94950000 4.57343700 0.00000000
H 13.28720000 2.54780200 0.00000000
H 13.40913800 5.52038300 0.00000000
H 14.58349300 4.52102000 0.89200000
H 14.58349300 4.52102000 -0.89200000
```

B3LYP-D3

Zero-point correction= 0.492138 (Hartree/Particle)
Thermal correction to Energy= 0.530510
Thermal correction to Enthalpy= 0.531454
Thermal correction to Gibbs Free Energy= 0.403664
Sum of electronic and zero-point Energies= -1704.175141
Sum of electronic and thermal Energies= -1704.136769
Sum of electronic and thermal Enthalpies= -1704.135825
Sum of electronic and thermal Free Energies= -1704.263615
Electronic energy:
E(RB3LYP)= -1704.667279

```
C -13.92199000 -3.45500400 0.00000000
C -12.50857300 -4.02007400 0.00000000
H -14.44955200 -3.83182000 0.88168800
H -13.96159200 -2.36108400 0.00000000
H -14.44955200 -3.83182000 -0.88168800
C -10.12115600 -3.50342500 0.00000000
H -9.89299600 -4.12275500 0.87847200
H -9.89299600 -4.12275500 -0.87847200
```

C -9.24798000 -2.24602600 0.00000000
C -6.95724900 -1.39713100 0.00000000
H -7.09177600 -0.75382800 -0.87989900
O -12.28759500 -5.22748400 0.00000000
N -11.50549500 -3.10185600 0.00000000
H -11.66831200 -2.10199500 0.00000000
O -9.73081200 -1.11379500 0.00000000
N -7.91666800 -2.47542200 0.00000000
H -7.51874900 -3.40942200 0.00000000
H -7.09177600 -0.75382800 0.87989900
C -5.55174500 -2.00745000 0.00000000
O -5.37360600 -3.22508800 0.00000000
N -4.54103800 -1.11365900 0.00000000
C -3.15678300 -1.52480800 0.00000000
H -4.69182100 -0.10930300 0.00000000
H -2.93236900 -2.14171100 0.88022500
H -2.93236900 -2.14171100 -0.88022500
C -2.28726500 -0.26256200 0.00000000
O -2.78325200 0.86387300 0.00000000
N -0.95728500 -0.48490200 0.00000000
C 0.00000000 0.59658200 0.00000000
H -0.55272600 -1.41665400 0.00000000
H -0.13579700 1.23851300 -0.88037100
H -0.13579700 1.23851300 0.88037100
C 1.40510000 -0.01621800 0.00000000
C 3.80111500 0.46017700 0.00000000
H 4.02410000 -0.15682200 0.88041400
H 4.02410000 -0.15682200 -0.88041400
C 4.67166900 1.72183900 0.00000000
C 6.95832000 2.58115100 0.00000000
H 6.82214900 3.22299900 -0.88036000
O 1.57778400 -1.23484700 0.00000000
N 2.41738000 0.87429500 0.00000000
H 2.26967400 1.87932300 0.00000000
O 4.17586800 2.84818900 0.00000000
N 6.00156300 1.49904600 0.00000000
H 6.40553500 0.56712900 0.00000000
H 6.82214900 3.22299900 0.88036000
C 8.36339800 1.96967300 0.00000000
O 8.53847000 0.75144000 0.00000000
N 9.37620000 2.86039300 0.00000000
C 10.75753100 2.44085100 0.00000000
H 9.23006100 3.86547300 0.00000000
H 10.97542000 1.82197300 0.88083700
H 10.97542000 1.82197300 -0.88083700
C 11.63774400 3.69746700 0.00000000
O 11.14326100 4.82143900 0.00000000
N 12.97193700 3.45170600 0.00000000
C 13.97379400 4.50704100 0.00000000
H 13.28539600 2.49068200 0.00000000
H 13.44841800 5.46266300 0.00000000
H 14.60653700 4.44528700 0.89233500
H 14.60653700 4.44528700 -0.89233500

Dimer 4

B3LYP

Zero-point correction= 1.001484 (Hartree/Particle)
Thermal correction to Energy= 1.072794
Thermal correction to Enthalpy= 1.073738
Thermal correction to Gibbs Free Energy= 0.881121
Sum of electronic and zero-point Energies= -3408.304632
Sum of electronic and thermal Energies= -3408.233322
Sum of electronic and thermal Enthalpies= -3408.232378
Sum of electronic and thermal Free Energies= -3408.424995
E(RB3LYP)= -3409.306116

C -0.24265300 -3.56570500 0.73351900
C 1.10843900 -3.38930100 0.01903700
H -0.32578000 -4.61901300 1.00190300
H -0.25368400 -2.95651800 1.64542100
C 2.65763900 -1.78871400 -1.00684400
H 2.69609200 -2.44731700 -1.88251700
H 2.56352300 -0.75209500 -1.32504700
C 3.96803200 -1.91668400 -0.21776100
C 5.58174700 -3.46381600 0.78272800
H 5.51776100 -4.49063300 1.14384600

O 1.79691600 -4.36474800 -0.30076400
N 1.48018700 -2.10940800 -0.21243400
H 0.85196500 -1.34536400 0.03665900
O 4.58595500 -0.93188600 0.20374600
N 4.40465600 -3.18253200 -0.02903600
H 3.75639600 -3.93235700 -0.27006100
H 5.60003100 -2.77644100 1.63632500
C 0.24265300 3.56570500 -0.73351900
H 0.32578000 4.61901300 -1.00190300
H 0.25368400 2.95651800 -1.64542100
C 3.06958600 1.73810500 1.06640800
H 3.01056200 2.35475900 1.97084600
H 3.13925200 0.68655600 1.33773800
C 6.05513900 3.55239000 -0.48008200
H 6.18453400 4.62275500 -0.64009600
N 1.38633700 3.20695400 0.09663500
H 2.04300000 3.92736100 0.39878600
C 1.78834800 1.92465700 0.24281800
O 1.16507000 0.96817100 -0.23645400
N 4.27727500 2.07927200 0.32957600
H 4.88211700 1.31364000 0.03111600
O 4.00072900 4.33132200 0.53866200
C 4.68047400 3.36140000 0.18369200
C 6.89872500 -3.34880200 -0.00343700
O 7.52916200 -4.36030600 -0.33356200
N 7.31086200 -2.09125900 -0.28618900
C 8.43758700 -1.82813100 -1.17697300
H 6.72188700 -1.29463500 -0.04145100
H 8.38652200 -2.51238400 -2.03285100
H 8.34202000 -0.80128400 -1.52603900
C 9.82218000 -1.96769100 -0.51425400
O 10.57957900 -0.99843200 -0.36878000
N 10.13972500 -3.22555600 -0.14598800
C 11.38051600 -3.56646200 0.52629000
H 9.40882900 -3.92910300 -0.26261000
H 11.19672900 -3.88608700 1.55940300
H 11.89393400 -4.37649200 -0.00318700
H 12.01722700 -2.68106400 0.53447000
H 6.06312700 3.03716100 -1.44854600
N 7.16544400 3.06757700 0.33322300
H 7.86968000 3.72454600 0.67053300
C 7.51609100 1.76240100 0.35141300
O 6.86743700 0.88422200 -0.23274900
C 8.78797900 1.44401000 1.14493600
H 8.76868000 0.38312100 1.39553500
H 8.81406400 2.03734200 2.06479900
C 10.47295500 2.96241200 0.23716100
O 9.89359100 3.94633100 0.71503800
C 11.77667000 3.09924600 -0.52626000
H 11.63175000 3.79521600 -1.35822400
H 12.52809100 3.53834500 0.13785300
H 12.15248800 2.14708800 -0.91077000
N 9.99178600 1.70318600 0.37436200
H 10.41902400 0.88968100 -0.06870600
C -11.77667000 -3.09924600 0.52626000
C -10.47295500 -2.96241200 -0.23716100
H -12.52809100 -3.53834500 -0.13785300
H -11.63175000 -3.79521600 1.35822400
H -12.15248800 -2.14708800 0.91077000
C -8.78797900 -1.44401000 -1.14493600
H -8.81406400 -2.03734200 -2.06479900
H -8.76868000 -0.38312100 -1.39553500
C -7.51609100 -1.76240100 -0.35141300
C -6.05513900 -3.55239000 0.48008200
H -6.18453400 -4.62275500 0.64009600
O -9.89359100 -3.94633100 -0.71503800
N -9.99178600 -1.70318600 -0.37436200
H -10.41902400 -0.88968100 0.06870600
O -6.86743700 -0.88422200 0.23274900
N -7.16544400 -3.06757700 -0.33322300
H -7.86968000 -3.72454600 -0.67053300
H -6.06312700 -3.03716100 1.44854600
C -11.38051600 3.56646200 -0.52629000
H -11.89393400 4.37649200 0.00318700
H -11.19672900 3.88608700 -1.55940300
H -12.01722700 2.68106400 -0.53447000
C -8.43758700 1.82813100 1.17697300
H -8.38652200 2.51238400 2.03285100
H -8.34202000 0.80128400 1.52603900
C -5.58174700 3.46381600 -0.78272800
H -5.51776100 4.49063300 -1.14384600
N -10.13972500 3.22555600 0.14598800
H -9.40882900 3.92910300 0.26261000
C -9.82218000 1.96769100 0.51425400

O -10.57957900 0.99843200 0.36878000
N -7.31086200 2.09125900 0.28618900
H -6.72188700 1.29463500 0.04145100
O -7.52916200 4.36030600 0.33356200
C -6.89872500 3.34880200 0.00343700
C -4.68047400 -3.36140000 -0.18369200
O -4.00072900 -4.33132200 -0.53866200
N -4.27727500 -2.07927200 -0.32957600
C -3.06958600 -1.73810500 -1.06640800
H -4.88211700 -1.31364000 -0.03111600
H -3.01056200 -2.35475900 -1.97084600
H -3.13925200 -0.68655600 -1.33773800
C -1.78834800 -1.92465700 -0.24281800
O -1.16507000 -0.96817100 0.23645400
N -1.38633700 -3.20695400 -0.09663500
H -2.04300000 -3.92736100 -0.39878600
H -5.60003100 2.77644100 -1.63632500
N -4.40465600 3.18253200 0.02903600
H -3.75639600 3.93235700 0.27006100
C -3.96803200 1.91668400 0.21776100
O -4.58595500 0.93188600 -0.20374600
C -2.65763900 1.78871400 1.00684400
H -2.56352300 0.75209500 1.32504700
H -2.69609200 2.44731700 1.88251700
C -1.10843900 3.38930100 -0.01903700
O -1.79691600 4.36474800 0.30076400
N -1.48018700 2.10940800 0.21243400
H -0.85196500 1.34536400 -0.03665900

B3LYP-D3

Zero-point correction= 1.003742 (Hartree/Particle)
Thermal correction to Energy= 1.074190
Thermal correction to Enthalpy= 1.075134
Thermal correction to Gibbs Free Energy= 0.882935
Sum of electronic and zero-point Energies= -3408.472894
Sum of electronic and thermal Energies= -3408.402446
Sum of electronic and thermal Enthalpies= -3408.401502
Sum of electronic and thermal Free Energies= -3408.593701
E(RB3LYP)= -3409.476636

C -0.17824500 -3.45235200 0.98281300
C 1.16567500 -3.31720900 0.24912700
H -0.26353700 -4.48865000 1.30995900
H -0.18618100 -2.78990200 1.85654300
C 2.70067700 -1.78751000 -0.88829700
H 2.72648400 -2.49641300 -1.72425100
H 2.61061700 -0.77047400 -1.26471500
C 4.00989000 -1.88219200 -0.09683900
C 5.63847800 -3.37218100 0.95353300
H 5.58283300 -4.37539200 1.37658900
O 1.84938500 -4.30981800 -0.02489000
N 1.53212000 -2.05315300 -0.06281300
H 0.91206700 -1.26984700 0.14230600
O 4.62376600 -0.88021600 0.29031400
N 4.45014100 -3.13602100 0.14506700
H 3.80494100 -3.89854300 -0.05946300
H 5.67367400 -2.63250000 1.76158200
C 0.17824500 3.45235200 -0.98281300
H 0.26353700 4.48865000 -1.30995900
H 0.18618100 2.78990200 -1.85654300
C 2.98332700 1.70014700 0.91432200
H 2.91379700 2.34735500 1.79623300
H 3.05305300 0.65888500 1.22192000
C 5.97628100 3.48497500 -0.63929100
H 6.13450700 4.55621300 -0.76383500
N 1.31757300 3.13303300 -0.13254400
H 1.96016700 3.86777000 0.16400800
C 1.71780700 1.85871200 0.06543400
O 1.10635200 0.88687500 -0.39890100
N 4.19440700 2.02280400 0.17666800
H 4.78541600 1.24552700 -0.11684500
O 3.90393100 4.27716100 0.32960700
C 4.59191900 3.30305400 0.00278100
C 6.93241100 -3.30336300 0.12754700
O 7.56330900 -4.33125800 -0.14487300
N 7.31968300 -2.06650500 -0.26336900
C 8.40509500 -1.86489200 -1.21975800

H 6.71751700 -1.26128000 -0.08669500
H 8.32179900 -2.60758900 -2.02302500
H 8.28873700 -0.86411900 -1.63223200
C 9.81493300 -1.95560200 -0.60543000
O 10.57389400 -0.97726400 -0.56572500
N 10.15274200 -3.17971900 -0.15425800
C 11.41671200 -3.46185500 0.50197000
H 9.42281700 -3.89272000 -0.18673100
H 11.27401000 -3.64577000 1.57414700
H 11.88798900 -4.34374300 0.05538400
H 12.07119000 -2.59867100 0.37353200
H 5.98541000 2.99743200 -1.62153500
N 7.05494500 2.93794100 0.17357000
H 7.72383300 3.56035300 0.62829000
C 7.38992100 1.63148800 0.11710100
O 6.77097500 0.81000500 -0.57326000
C 8.60014900 1.23476300 0.96737700
H 8.57905500 0.15217100 1.09546800
H 8.54292400 1.72055300 1.94740400
C 10.32025200 2.86153600 0.36246100
O 9.69168400 3.78484100 0.89705600
C 11.67207900 3.09392000 -0.28490400
H 11.56269900 3.84824200 -1.07028300
H 12.35267500 3.50369000 0.46790500
H 12.10995300 2.18678300 -0.71091600
N 9.85271000 1.59178200 0.32646500
H 10.33041000 0.83232100 -0.15951300
C -11.67207900 -3.09392000 0.28490400
C -10.32025200 -2.86153600 -0.36246100
H -12.35267500 -3.50369000 -0.46790500
H -11.56269900 -3.84824200 1.07028300
H -12.10995300 -2.18678300 0.71091600
C -8.60014900 -1.23476300 -0.96737700
H -8.54292400 -1.72055300 -1.94740400
H -8.57905500 -0.15217100 -1.09546800
C -7.38992100 -1.63148800 -0.11710100
C -5.97628100 -3.48497500 0.63929100
H -6.13450700 -4.55621300 0.76383500
O -9.69168400 -3.78484100 -0.89705600
N -9.85271000 -1.59178200 -0.32646500
H -10.33041000 -0.83232100 0.15951300
O -6.77097500 -0.81000500 0.57326000
N -7.05494500 -2.93794100 -0.17357000
H -7.72383300 -3.56035300 -0.62829000
H -5.98541000 -2.99743200 1.62153500
C -11.41671200 3.46185500 -0.50197000
H -11.88798900 4.34374300 -0.05538400
H -11.27401000 3.64577000 -1.57414700
H -12.07119000 2.59867100 -0.37353200
C -8.40509500 1.86489200 1.21975800
H -8.32179900 2.60758900 2.02302500
H -8.28873700 0.86411900 1.63223200
C -5.63847800 3.37218100 -0.95353300
H -5.58283300 4.37539200 -1.37658900
N -10.15274200 3.17971900 0.15425800
H -9.42281700 3.89272000 0.18673100
C -9.81493300 1.95560200 0.60543000
O -10.57389400 0.97726400 0.56572500
N -7.31968300 2.06650500 0.26336900
H -6.71751700 1.26128000 0.08669500
O -7.56330900 4.33125800 0.14487300
C -6.93241100 3.30336300 -0.12754700
C -4.59191900 -3.30305400 -0.00278100
O -3.90393100 -4.27716100 -0.32960700
N -4.19440700 -2.02280400 -0.17666800
C -2.98332700 -1.70014700 -0.91432200
H -4.78541600 -1.24552700 0.11684500
H -2.91379700 -2.34735500 -1.79623300
H -3.05305300 -0.65888500 -1.22192000
C -1.71780700 -1.85871200 -0.06543400
O -1.10635200 -0.88687500 0.39890100
N -1.31757300 -3.13303300 0.13254400
H -1.96016700 -3.86777000 -0.16400800
H -5.67367400 2.63250000 -1.76158200
N -4.45014100 3.13602100 -0.14506700
H -3.80494100 3.89854300 0.05946300
C -4.00989000 1.88219200 0.09683900
O -4.62376600 0.88021600 -0.29031400
C -2.70067700 1.78751000 0.88829700
H -2.61061700 0.77047400 1.26471500
H -2.72648400 2.49641300 1.72425100
C -1.16567500 3.31720900 -0.24912700
O -1.84938500 4.30981800 0.02489000
N -1.53212000 2.05315300 0.06281300

H -0.91206700 1.26984700 -0.14230600

B3LYP

Monomer 6

Zero-point correction= 0.714982 (Hartree/Particle)
Thermal correction to Energy= 0.771274
Thermal correction to Enthalpy= 0.772218
Thermal correction to Gibbs Free Energy= 0.596044
Sum of electronic and zero-point Energies= -2535.949881
Sum of electronic and thermal Energies= -2535.893588
Sum of electronic and thermal Enthalpies= -2535.892644
Sum of electronic and thermal Free Energies= -2536.068819
E(RB3LYP)= -2536.664862

```
C -1.40604500 -0.00001000 0.00000000
O -1.57066400 -1.22010600 0.00000000
N -2.42077200 0.88673200 0.00000000
C -3.80752100 0.48092400 0.00000000
H -2.27831000 1.89335500 0.00000000
H -4.03692800 -0.13292900 0.88087700
H -4.03692800 -0.13292900 -0.88087700
C -4.66129900 1.75538600 0.00000000
O -4.14248900 2.87185300 0.00000000
N -5.99401400 1.55544800 0.00000000
C -6.94307600 2.64498400 0.00000000
H -6.41655300 0.63079900 0.00000000
H -6.80528900 3.28568200 -0.88089100
H -6.80528900 3.28568200 0.88089100
C -8.34919800 2.03215600 0.00000000
C -10.75032300 2.51184700 0.00000000
H -10.97905700 1.89763800 0.88086400
H -10.97905700 1.89763800 -0.88086400
C -11.60585800 3.78474400 0.00000000
C -13.88731000 4.67284900 0.00000000
H -13.74907600 5.31368800 -0.88076200
O -8.51463600 0.81223200 0.00000000
N -9.36404800 2.91906500 0.00000000
H -9.22150100 3.92559200 0.00000000
O -11.08982500 4.90220700 0.00000000
N -12.93886500 3.58294600 0.00000000
H -13.35950900 2.65770300 0.00000000
H -13.74907600 5.31368800 0.88076200
C -15.29374700 4.06270500 0.00000000
O -15.46376000 2.84358900 0.00000000
N -16.30855500 4.95102400 0.00000000
C -17.69239300 4.53872300 0.00000000
H -16.16642000 5.95730400 0.00000000
H -17.91660400 3.92264900 0.88124800
H -17.91660400 3.92264900 -0.88124800
C -18.55792700 5.80569400 0.00000000
O -18.04621100 6.92211700 0.00000000
N -19.89550300 5.58120800 0.00000000
C -20.87928600 6.65332400 0.00000000
H -20.22423700 4.62525200 0.00000000
H -20.33568500 7.59840400 0.00000000
H -21.51339800 6.60303500 0.89202900
H -21.51339800 6.60303500 -0.89202900
C 0.00000000 0.61339400 0.00000000
H 0.13744100 1.25411200 -0.88094600
N 0.94935000 -0.47586100 0.00000000
H 0.13744100 1.25411200 0.88094600
C 2.28207700 -0.27599300 0.00000000
H 0.52675900 -1.40052400 0.00000000
C 3.13561700 -1.55055500 0.00000000
O 2.80092400 0.84050800 0.00000000
H 2.90585600 -2.16439900 0.88082500
H 2.90585600 -2.16439900 -0.88082500
N 4.52248100 -1.14548500 0.00000000
C 5.53671700 -2.03313700 0.00000000
H 4.66573500 -0.13903000 0.00000000
C 6.94297100 -1.42071300 0.00000000
O 5.37050300 -3.25298300 0.00000000
N 7.89255800 -2.50948500 0.00000000
H 7.08040800 -0.77968800 -0.88078700
H 7.08040800 -0.77968800 0.88078700
C 9.22561000 -2.30804100 0.00000000
```

H 7.47130400 -3.43459900 0.00000000
C 10.07992500 -3.58157200 0.00000000
O 9.74179800 -1.19040200 0.00000000
N 11.46681300 -3.17815900 0.00000000
H 9.84937200 -4.19566200 0.88061600
H 9.84937200 -4.19566200 -0.88061600
C 12.47965000 -4.06933900 0.00000000
H 11.61333800 -2.17250800 0.00000000
C 13.88627100 -3.45977700 0.00000000
O 12.30705100 -5.28799300 0.00000000
H 14.02235900 -2.81728700 -0.88027400
N 14.83807300 -4.54518300 0.00000000
H 14.02235900 -2.81728700 0.88027400
C 16.17265400 -4.33577500 0.00000000
H 14.42422500 -5.47289900 0.00000000
C 17.03140600 -5.60381300 0.00000000
O 16.67445200 -3.21163900 0.00000000
H 16.79718900 -6.22037800 0.87889800
H 16.79718900 -6.22037800 -0.87889800
N 18.41797000 -5.20971400 0.00000000
C 19.42382900 -6.12506800 0.00000000
H 18.57833400 -4.20902800 0.00000000
C 20.83367200 -5.55084000 0.00000000
O 19.20862900 -7.33356000 0.00000000
H 21.36609900 -5.92141200 0.88153700
H 20.86269600 -4.45679300 0.00000000
H 21.36609900 -5.92141200 -0.88153700

B3LYP

Dimer 6

Zero-point correction= 1.454779 (Hartree/Particle)
Thermal correction to Energy= 1.558717
Thermal correction to Enthalpy= 1.559661
Thermal correction to Gibbs Free Energy= 1.291570
Sum of electronic and zero-point Energies= -5071.984464
Sum of electronic and thermal Energies= -5071.880526
Sum of electronic and thermal Enthalpies= -5071.879582
Sum of electronic and thermal Free Energies= -5072.147673
E(RB3LYP)= -5073.439243

C 11.82660100 3.57093500 0.48209500
C 10.45480100 3.37870200 -0.18703500
H 11.96791600 4.64410400 0.60965100
H 11.81507800 3.08792600 1.46713800
C 8.84324300 1.75974800 -1.07760100
H 8.77698000 2.38937500 -1.97244300
H 8.91596500 0.71264200 -1.36480900
C 7.56551100 1.92928000 -0.24486200
C 6.04985600 3.54928200 0.80928000
H 6.14678500 4.59265700 1.10986600
O 9.77274700 4.35026600 -0.53413900
N 10.05335900 2.09735000 -0.34298100
H 10.64873200 1.33014700 -0.02913000
O 6.93549300 0.96258200 0.20481900
N 7.17575400 3.20887500 -0.05203300
H 7.83066300 3.93471000 -0.34614300
H 6.07360600 2.91133700 1.70119800
C 11.40558700 -3.41323500 -0.87258400
H 11.37187900 -4.42217500 -1.28482700
H 11.46504100 -2.68542100 -1.69023100
C 8.38453800 -1.84477400 0.85227200
H 8.37558500 -2.56822800 1.67601900
H 8.28504400 -0.83415000 1.24407000
C 5.55530800 -3.44409900 -1.15184700
H 5.49294200 -4.45915200 -1.54452000
N 10.18329000 -3.18000200 -0.11377000
H 9.52236400 -3.94135700 0.03986800
C 9.72938700 -1.92704100 0.11725600
O 10.35889500 -0.91905600 -0.22441300
N 7.24366200 -2.08764200 -0.01878400
H 6.62408200 -1.30241700 -0.21753500
O 7.54673500 -4.34487900 -0.10496600
C 6.87762400 -3.34103300 -0.37230300
C 4.68089000 3.40693500 0.12201700
O 3.98628500 4.39814700 -0.13144700
N 4.29746500 2.14020400 -0.15587800
C 3.09120500 1.85591900 -0.92060600

H 4.92884700 1.36432200 0.04443400
H 3.02201000 2.55436400 -1.76305500
H 3.17238200 0.83536500 -1.28947000
C 1.80976700 1.94979800 -0.08007700
O 1.19079100 0.94558400 0.29638300
N 1.40429700 3.20757600 0.20293700
C 0.26882000 3.47296600 1.07813900
H 2.05635300 3.95867200 -0.02706600
H 5.56506000 -2.73136400 -1.98516600
N 4.38326500 -3.19595300 -0.32083100
H 3.72365700 -3.95120500 -0.13118200
C 3.96310700 -1.94331900 -0.03525700
O 4.58728100 -0.93178500 -0.38163400
C 2.66057100 -1.86500900 0.77351900
H 2.57887700 -0.85565600 1.17215300
H 2.70169700 -2.58997400 1.59513400
C 1.08956100 -3.36608900 -0.36424900
O 1.77410600 -4.36957900 -0.13449900
C -0.26882000 -3.47296600 -1.07813900
N 1.47246800 -2.11231900 -0.03106000
H 0.84043100 -1.32936900 -0.19799900
C 16.25067600 2.92600800 -0.18786600
O 15.67873400 3.88708100 -0.71703100
N 15.75376600 1.66594600 -0.25278300
C 14.55839500 1.37395700 -1.02430600
H 16.17470500 0.87312300 0.23250800
H 14.59805300 1.92104200 -1.97224500
H 14.53637400 0.30206300 -1.22302500
C 13.27767000 1.73618300 -0.26435000
O 12.60698100 0.88713600 0.33819800
N 12.94165800 3.04475000 -0.29830000
H 13.65576000 3.68132900 -0.65377400
N 15.90715800 -3.22998900 0.39278700
H 15.17401900 -3.93969900 0.42913300
C 15.55162300 -1.97623200 0.73423700
O 16.31424200 -1.00692800 0.62135200
C 14.13037400 -1.84376700 1.31461300
H 14.01748200 -0.82430100 1.68019100
H 14.02082000 -2.54726000 2.14922400
C 12.67288600 -3.32587100 -0.00470500
O 13.28361400 -4.34769000 0.32993900
N 13.06263100 -2.07789300 0.34552400
H 12.48220000 -1.27502400 0.10006800
C -11.40558700 3.41323500 0.87258400
C -12.67288600 3.32587100 0.00470500
H -11.37187900 4.42217500 1.28482700
H -11.46504100 2.68542100 1.69023100
C -14.13037400 1.84376700 -1.31461300
H -14.02082000 2.54726000 -2.14922400
H -14.01748200 0.82430100 -1.68019100
C -15.55162300 1.97623200 -0.73423700
O -13.28361400 4.34769000 -0.32993900
N -13.06263100 2.07789300 -0.34552400
H -12.48220000 1.27502400 -0.10006800
O -16.31424200 1.00692800 -0.62135200
N -15.90715800 3.22998900 -0.39278700
H -15.17401900 3.93969900 -0.42913300
C -11.82660100 -3.57093500 -0.48209500
H -11.96791600 -4.64410400 -0.60965100
H -11.81507800 -3.08792600 -1.46713800
C -14.55839500 -1.37395700 1.02430600
H -14.59805300 -1.92104200 1.97224500
H -14.53637400 -0.30206300 1.22302500
N -12.94165800 -3.04475000 0.29830000
H -13.65576000 -3.68132900 0.65377400
C -13.27767000 -1.73618300 0.26435000
O -12.60698100 -0.88713600 -0.33819800
N -15.75376600 -1.66594600 0.25278300
H -16.17470500 -0.87312300 -0.23250800
O -15.67873400 -3.88708100 0.71703100
C -16.25067600 -2.92600800 0.18786600
C -1.08956100 3.36608900 0.36424900
C -2.66057100 1.86500900 -0.77351900
H -2.70169700 2.58997400 -1.59513400
H -2.57887700 0.85565600 -1.17215300
C -3.96310700 1.94331900 0.03525700
C -5.55530800 3.44409900 1.15184700
H -5.49294200 4.45915200 1.54452000
O -1.77410600 4.36957900 0.13449900
N -1.47246800 2.11231900 0.03106000
H -0.84043100 1.32936900 0.19799900
O -4.58728100 0.93178500 0.38163400
N -4.38326500 3.19595300 0.32083100
H -3.72365700 3.95120500 0.13118200

```
H -5.56506000 2.73136400 1.98516600
C -3.09120500 -1.85591900 0.92060600
H -3.02201000 -2.55436400 1.76305500
H -3.17238200 -0.83536500 1.28947000
C -6.04985600 -3.54928200 -0.80928000
H -6.14678500 -4.59265700 -1.10986600
N -1.40429700 -3.20757600 -0.20293700
H -2.05635300 -3.95867200 0.02706600
C -1.80976700 -1.94979800 0.08007700
O -1.19079100 -0.94558400 -0.29638300
N -4.29746500 -2.14020400 0.15587800
H -4.92884700 -1.36432200 -0.04443400
O -3.98628500 -4.39814700 0.13144700
C -4.68089000 -3.40693500 -0.12201700
C -6.87762400 3.34103300 0.37230300
O -7.54673500 4.34487900 0.10496600
N -7.24366200 2.08764200 0.01878400
C -8.38453800 1.84477400 -0.85227200
H -6.62408200 1.30241700 0.21753500
H -8.37558500 2.56822800 -1.67601900
H -8.28504400 0.83415000 -1.24407000
C -9.72938700 1.92704100 -0.11725600
O -10.35889500 0.91905600 0.22441300
N -10.18329000 3.18000200 0.11377000
H -9.52236400 3.94135700 -0.03986800
H -6.07360600 -2.91133700 -1.70119800
N -7.17575400 -3.20887500 0.05203300
H -7.83066300 -3.93471000 0.34614300
C -7.56551100 -1.92928000 0.24486200
O -6.93549300 -0.96258200 -0.20481900
C -8.84324300 -1.75974800 1.07760100
H -8.91596500 -0.71264200 1.36480900
H -8.77698000 -2.38937500 1.97244300
C -10.45480100 -3.37870200 0.18703500
O -9.77274700 -4.35026600 0.53413900
N -10.05335900 -2.09735000 0.34298100
H -10.64873200 -1.33014700 0.02913000
H -0.35241700 -4.49335400 -1.45268800
H -0.29222600 -2.77308400 -1.92221800
H 0.29222600 2.77308400 1.92221800
H 0.35241700 4.49335400 1.45268800
C -17.19687300 3.52628200 0.20718300
H -17.37419700 4.60267400 0.15029900
H -17.98575600 2.99973400 -0.33593800
H -17.24034900 3.21397300 1.25853000
C -17.57509600 -3.07568100 -0.53761100
H -17.65032900 -4.08901500 -0.93761500
H -18.39778700 -2.92597700 0.17253000
H -17.69009800 -2.34632500 -1.34598100
C 17.57509600 3.07568100 0.53761100
H 18.39778700 2.92597700 -0.17253000
H 17.65032900 4.08901500 0.93761500
H 17.69009800 2.34632500 1.34598100
C 17.19687300 -3.52628200 -0.20718300
H 17.98575600 -2.99973400 0.33593800
H 17.37419700 -4.60267400 -0.15029900
H 17.24034900 -3.21397300 -1.25853000
```

B3LYP

Monomer 8

Zero-point correction= 0.937658 (Hartree/Particle)
Thermal correction to Energy= 1.012070
Thermal correction to Enthalpy= 1.013014
Thermal correction to Gibbs Free Energy= 0.786366
Sum of electronic and zero-point Energies= -3367.779303
Sum of electronic and thermal Energies= -3367.704891
Sum of electronic and thermal Enthalpies= -3367.703947
Sum of electronic and thermal Free Energies= -3367.930595
E(RB3LYP)= -3368.716961

```
C -0.52945900 -28.78594400 0.00000000
C 0.41806800 -27.59446300 0.00000000
H -0.32355700 -29.40112200 0.88151300
H -1.58751300 -28.50611800 0.00000000
H -0.32355700 -29.40112200 -0.88151300
C 0.59034300 -25.15190200 0.00000000
H 1.24782500 -25.10037100 0.87889300
```

H 1.24782500 -25.10037100 -0.87889300
C -0.38552400 -23.97146500 0.00000000
C -0.58384100 -21.53098600 0.00000000
H -1.23862600 -21.48106700 -0.88029400
O 1.63829800 -27.72781200 0.00000000
N -0.17751400 -26.37181300 0.00000000
H -1.18292500 -26.24439100 0.00000000
O -1.60530900 -24.13743900 0.00000000
N 0.19033300 -22.74947500 0.00000000
H 1.19694500 -22.61286000 0.00000000
H -1.23862600 -21.48106700 0.88029400
C 0.39668200 -20.35241900 0.00000000
O 1.61466900 -20.52952400 0.00000000
N -0.17386800 -19.12995500 0.00000000
C 0.60283100 -17.91218500 0.00000000
H -1.18017400 -18.98792000 0.00000000
H 1.25689600 -17.86337800 0.88063000
H 1.25689600 -17.86337800 -0.88063000
C -0.37979000 -16.73465400 0.00000000
O -1.59734900 -16.91655000 0.00000000
N 0.18777400 -15.51182000 0.00000000
C -0.59032500 -14.29457000 0.00000000
H 1.19395600 -15.36720400 0.00000000
H -1.24407200 -14.24639000 -0.88079500
H -1.24407200 -14.24639000 0.88079500
C 0.39287100 -13.11713300 0.00000000
C 0.60431900 -10.67696500 0.00000000
H 1.25790000 -10.62876100 0.88086400
H 1.25790000 -10.62876100 -0.88086400
C -0.37976300 -9.50009100 0.00000000
C -0.59273500 -7.05992500 0.00000000
H -1.24624100 -7.01193200 -0.88090600
O 1.61020300 -13.30082200 0.00000000
N -0.17386800 -11.89434600 0.00000000
H -1.18003200 -11.74889800 0.00000000
O -1.59693700 -9.68527900 0.00000000
N 0.18595200 -8.27707300 0.00000000
H 1.19205700 -8.13078200 0.00000000
H -1.24624100 -7.01193200 0.88090600
C 0.39140900 -5.88298600 0.00000000
O 1.60853900 -6.06856800 0.00000000
N -0.17413600 -4.66001000 0.00000000
C 0.60457700 -3.44281900 0.00000000
H -1.18023800 -4.51351100 0.00000000
H 1.25803500 -3.39482800 0.88092500
H 1.25803500 -3.39482800 -0.88092500
C -0.37979000 -2.26602800 0.00000000
O -1.59687800 -2.45198800 0.00000000
N 0.18553500 -1.04301000 0.00000000
H 1.19163300 -0.89636600 0.00000000
C -0.59320800 0.17417900 0.00000000
C 0.39129300 1.35089100 0.00000000
C 0.60527500 3.79098300 0.00000000
H 1.25871600 3.83878700 0.88094000
H 1.25871600 3.83878700 -0.88094000
C -0.37888900 4.96795300 0.00000000
C -0.59212100 7.40811500 0.00000000
H -1.24555700 7.45612300 -0.88093600
O 1.60835000 1.16471600 0.00000000
N -0.17380300 2.57399200 0.00000000
H -1.17986800 2.72087100 0.00000000
O -1.59598800 4.78214900 0.00000000
N 0.18659300 6.19090000 0.00000000
H 1.19270300 6.33739900 0.00000000
H -1.24555700 7.45612300 0.88093600
C 0.39235600 8.58479100 0.00000000
O 1.60940600 8.39874500 0.00000000
N -0.17268200 9.80799300 0.00000000
C 0.60685000 11.02466600 0.00000000
H -1.17871400 9.95489500 0.00000000
H 1.26034200 11.07218400 0.88092900
H 1.26034200 11.07218400 -0.88092900
C -0.37659700 12.20211100 0.00000000
O -1.59377900 12.01734200 0.00000000
N 0.18954700 13.42493800 0.00000000
C -0.58926100 14.64204200 0.00000000
H 1.19567800 13.57080900 0.00000000
H -1.24277900 14.68993700 -0.88090500
H -1.24277900 14.68993700 0.88090500
C 0.39454600 15.81901500 0.00000000
C 0.60951800 18.25824900 0.00000000
H 1.26318700 18.30509000 0.88088700
H 1.26318700 18.30509000 -0.88088700
C -0.37190400 19.43694900 0.00000000

C -0.58333400 21.87609000 0.00000000
H -1.23714300 21.92350400 -0.88078400
O 1.61168700 15.63424800 0.00000000
N -0.17097800 17.04232700 0.00000000
H -1.17696700 17.18874200 0.00000000
O -1.58928600 19.25535800 0.00000000
N 0.19615400 20.65957000 0.00000000
H 1.20230800 20.80343100 0.00000000
H -1.23714300 21.92350400 0.88078400
C 0.39767000 23.05432600 0.00000000
O 1.61537300 22.87462300 0.00000000
N -0.16958700 24.27787600 0.00000000
C 0.61481000 25.49017100 0.00000000
H -1.17526100 24.42426600 0.00000000
H 1.26899900 25.53228500 0.88124500
H 1.26899900 25.53228500 -0.88124500
C -0.35831200 26.67663500 0.00000000
O -1.57345100 26.49881700 0.00000000
N 0.23259300 27.89741100 0.00000000
C -0.52017300 29.14261400 0.00000000
H 1.24239500 27.94456700 0.00000000
H -1.57985000 28.88617900 0.00000000
H -0.29387900 29.73713400 0.89199900
H -0.29387900 29.73713400 -0.89199900
H -1.24664400 0.22218000 -0.88093600
H -1.24664400 0.22218000 0.88093600

B3LYP

Dimer 8

Zero-point correction= 1.908888 (Hartree/Particle)
Thermal correction to Energy= 2.045272
Thermal correction to Enthalpy= 2.046216
Thermal correction to Gibbs Free Energy= 1.703427
Sum of electronic and zero-point Energies= -6735.665247
Sum of electronic and thermal Energies= -6735.528863
Sum of electronic and thermal Enthalpies= -6735.527919
Sum of electronic and thermal Free Energies= -6735.870708
E(RB3LYP)= -6737.574135
C 11.81178700 3.55458700 0.79894400
C 10.46005000 3.39161700 0.08299600
H 11.89667400 4.60363700 1.08297400
H 11.82163000 2.93189800 1.70170400
C 8.89632100 1.80763800 -0.94932600
H 8.85610900 2.47260700 -1.82017700
H 8.98335400 0.77307600 -1.27543800
C 7.59130300 1.93922900 -0.15154600
C 6.02087100 3.51208500 0.89261600
H 6.09474900 4.54882100 1.22151300
O 9.77682900 4.37456500 -0.22632900
N 10.08022000 2.11640900 -0.15952400
H 10.70577000 1.34699700 0.07964200
O 6.96187900 0.95361300 0.25502900
N 7.17753400 3.20858800 0.05892900
H 7.83985700 3.94780100 -0.17987100
H 6.02351000 2.85186300 1.76835100
C 11.34191500 -3.49814800 -0.89793600
H 11.27517200 -4.53178400 -1.23786900
H 11.34812400 -2.82926300 -1.76708700
C 8.43964400 -1.81967500 0.92778100
H 8.46288300 -2.50690400 1.78175400
H 8.35618800 -0.79304200 1.27918000
C 5.54735400 -3.49748900 -0.92212300
H 5.47608200 -4.53034800 -1.26351800
N 10.17404200 -3.20625200 -0.07516200
H 9.50755500 -3.94843900 0.14036200
C 9.75355200 -1.94012200 0.14265700
O 10.38311500 -0.94857700 -0.24846500
N 7.26508800 -2.09799300 0.11332100
H 6.64393800 -1.31909800 -0.10483800
O 7.55726800 -4.35857500 0.11887100
C 6.88467100 -3.36361200 -0.17419100
C 4.68007600 3.37113900 0.15230200
O 4.00431500 4.36341800 -0.14309000
N 4.30008800 2.10332600 -0.12596400
C 3.12200800 1.81777700 -0.93301200

H 4.92188300 1.32634200 0.09734100
H 3.09264500 2.50104100 -1.78997500
H 3.20640700 0.78979400 -1.28005300
C 1.81180700 1.93926800 -0.14190400
O 1.18111700 0.94872200 0.25016500
N 1.39616100 3.20594800 0.08122500
C 0.23699000 3.49874400 0.91544800
H 2.06230100 3.94724900 -0.13934800
H 5.54734300 -2.82689200 -1.78997600
N 4.38692000 -3.20594400 -0.08905400
H 3.71985600 -3.94733800 0.12810200
C 3.97116600 -1.93960700 0.13599200
O 4.60203800 -0.94829400 -0.25377500
C 2.66029500 -1.81946900 0.92622900
H 2.57748300 -0.79297700 1.27808200
H 2.68708800 -2.50687100 1.77994700
C 1.10170200 -3.36427600 -0.17018500
O 1.77497600 -4.35926800 0.12162200
C -0.23699000 -3.49874400 -0.91544800
N 1.48295100 -2.09864000 0.11596100
H 0.86248500 -1.31941600 -0.10301500
C 23.33408800 3.10534200 0.66173000
C 22.03773700 2.97596900 -0.11536000
H 24.09058100 3.55471800 0.01039000
H 23.18006700 3.78996100 1.50144900
H 23.70844200 2.14897500 1.03711400
C 20.36366700 1.46636800 -1.05751400
H 20.39763700 2.07064400 -1.96997300
H 20.34822000 0.40851000 -1.32078600
C 19.08382900 1.77373100 -0.27242300
C 17.61359100 3.55166800 0.56805400
H 17.73982400 4.62035300 0.74122800
O 21.46121300 3.96487400 -0.58635000
N 21.55983300 1.71783100 -0.27261800
H 21.98420600 0.89957600 0.16432300
O 18.43056300 0.88788400 0.29477200
N 18.73128400 3.07814800 -0.24171500
H 19.43830700 3.73993800 -0.56356800
H 17.61469400 3.02560600 1.53074600
C 22.95080400 -3.54768500 -0.48682400
H 23.45620100 -4.37181600 0.02826900
H 22.77621400 -3.84201900 -1.52905800
H 23.59039900 -2.66452900 -0.46802900
C 19.99207900 -1.83867800 1.21839600
H 19.93249700 -2.53902300 2.06054900
H 19.89399000 -0.81848400 1.58585400
C 17.15695800 -3.43364800 -0.80374200
H 17.10024100 -4.45105300 -1.19167300
N 21.70483800 -3.21871200 0.18189200
H 20.97300600 -3.92412000 0.27965500
C 21.38221200 -1.96655800 0.56508700
O 22.13869700 -0.99399000 0.43869900
N 18.87397300 -2.08387800 0.31190000
H 18.28780100 -1.28227500 0.07699700
O 19.09004800 -4.35364600 0.31858100
C 18.46438400 -3.33537700 0.00051100
C 16.24424200 3.36623300 -0.10812900
O 15.56567000 4.33958600 -0.45639500
N 15.84366400 2.08552900 -0.27177500
C 14.64086700 1.75082100 -1.01961800
H 16.44646100 1.31733700 0.02432000
H 14.58615200 2.37787300 -1.91716200
H 14.71373200 0.70262300 -1.30266400
C 13.35472900 1.92630000 -0.20127100
O 12.72629400 0.96368700 0.25877300
N 12.95505300 3.20688200 -0.03606500
H 13.61471000 3.93057800 -0.32432000
H 17.18307800 -2.72460000 -1.63928500
N 15.96949800 -3.17745900 0.00155900
H 15.31455800 -3.93315300 0.20253500
C 15.52818600 -1.91791500 0.21974500
O 16.15076000 -0.92079400 -0.16397100
C 14.20450300 -1.81356800 0.99015300
H 14.11069700 -0.78984500 1.34792200
H 14.22197800 -2.50686000 1.83927100
C 12.67251800 -3.36021300 -0.13844100
O 13.34909300 -4.35193300 0.15550800
N 13.04146100 -2.09359900 0.16004600
H 12.42050500 -1.31647800 -0.06506200
C -11.34191500 3.49814800 0.89793600
C -12.67251800 3.36021300 0.13844100
H -11.27517200 4.53178400 1.23786900
H -11.34812400 2.82926300 1.76708700
C -14.20450300 1.81356800 -0.99015300

H -14.22197800 2.50686000 -1.83927100
H -14.11069700 0.78984500 -1.34792200
C -15.52818600 1.91791500 -0.21974500
C -17.15695800 3.43364800 0.80374200
H -17.10024100 4.45105300 1.19167300
O -13.34909300 4.35193300 -0.15550800
N -13.04146100 2.09359900 -0.16004600
H -12.42050500 1.31647800 0.06506200
O -16.15076000 0.92079400 0.16397100
N -15.96949800 3.17745900 -0.00155900
H -15.31455800 3.93315300 -0.20253500
H -17.18307800 2.72460000 1.63928500
C -11.81178700 -3.55458700 -0.79894400
H -11.89667400 -4.60363700 -1.08297400
H -11.82163000 -2.93189800 -1.70170400
C -14.64086700 -1.75082100 1.01961800
H -14.58615200 -2.37787300 1.91716200
H -14.71373200 -0.70262300 1.30266400
C -17.61359100 -3.55166800 -0.56805400
H -17.73982400 -4.62035300 -0.74122800
N -12.95505300 -3.20688200 0.03606500
H -13.61471000 -3.93057800 0.32432000
C -13.35472900 -1.92630000 0.20127100
O -12.72629400 -0.96368700 -0.25877300
N -15.84366400 -2.08552900 0.27177500
H -16.44646100 -1.31733700 -0.02432000
O -15.56567000 -4.33958600 0.45639500
C -16.24424200 -3.36623300 0.10812900
C -18.46438400 3.33537700 -0.00051100
O -19.09004800 4.35364600 -0.31858100
N -18.87397300 2.08387800 -0.31190000
C -19.99207900 1.83867800 -1.21839600
H -18.28780100 1.28227500 -0.07699700
H -19.93249700 2.53902300 -2.06054900
H -19.89399000 0.81848400 -1.58585400
C -21.38221200 1.96655800 -0.56508700
O -22.13869700 0.99399000 -0.43869900
N -21.70483800 3.21871200 -0.18189200
C -22.95080400 3.54768500 0.48682400
H -20.97300600 3.92412000 -0.27965500
H -22.77621400 3.84201900 1.52905800
H -23.45620100 4.37181600 -0.02826900
H -23.59039900 2.66452900 0.46802900
H -17.61469400 -3.02560600 -1.53074600
N -18.73128400 -3.07814800 0.24171500
H -19.43830700 -3.73993800 0.56356800
C -19.08382900 -1.77373100 0.27242300
O -18.43056300 -0.88788400 -0.29477200
C -20.36366700 -1.46636800 1.05751400
H -20.34822000 -0.40851000 1.32078600
H -20.39763700 -2.07064400 1.96997300
C -22.03773700 -2.97596900 0.11536000
O -21.46121300 -3.96487400 0.58635000
C -23.33408800 -3.10534200 -0.66173000
H -23.18006700 -3.78996100 -1.50144900
H -24.09058100 -3.55471800 -0.01039000
H -23.70844200 -2.14897500 -1.03711400
N -21.55983300 -1.71783100 0.27261800
H -21.98420600 -0.89957600 -0.16432300
C -1.10170200 3.36427600 0.17018500
C -2.66029500 1.81946900 -0.92622900
H -2.68708800 2.50687100 -1.77994700
H -2.57748300 0.79297700 -1.27808200
C -3.97116600 1.93960700 -0.13599200
C -5.54735400 3.49748900 0.92212300
H -5.47608200 4.53034800 1.26351800
O -1.77497600 4.35926800 -0.12162200
N -1.48295100 2.09864000 -0.11596100
H -0.86248500 1.31941600 0.10301500
O -4.60203800 0.94829400 0.25377500
N -4.38692000 3.20594400 0.08905400
H -3.71985600 3.94733800 -0.12810200
H -5.54734300 2.82689200 1.78997600
C -3.12200800 -1.81777700 0.93301200
H -3.09264500 -2.50104100 1.78997500
H -3.20640700 -0.78979400 1.28005300
C -6.02087100 -3.51208500 -0.89261600
H -6.09474900 -4.54882100 -1.22151300
N -1.39616100 -3.20594800 -0.08122500
H -2.06230100 -3.94724900 0.13934800
C -1.81180700 -1.93926800 0.14190400
O -1.18111700 -0.94872200 -0.25016500
N -4.30008800 -2.10332600 0.12596400
H -4.92188300 -1.32634200 -0.09734100

```
O -4.00431500 -4.36341800 0.14309000
C -4.68007600 -3.37113900 -0.15230200
C -6.88467100 3.36361200 0.17419100
O -7.55726800 4.35857500 -0.11887100
N -7.26508800 2.09799300 -0.11332100
C -8.43964400 1.81967500 -0.92778100
H -6.64393800 1.31909800 0.10483800
H -8.46288300 2.50690400 -1.78175400
H -8.35618800 0.79304200 -1.27918000
C -9.75355200 1.94012200 -0.14265700
O -10.38311500 0.94857700 0.24846500
N -10.17404200 3.20625200 0.07516200
H -9.50755500 3.94843900 -0.14036200
H -6.02351000 -2.85186300 -1.76835100
N -7.17753400 -3.20858800 -0.05892900
H -7.83985700 -3.94780100 0.17987100
C -7.59130300 -1.93922900 0.15154600
O -6.96187900 -0.95361300 -0.25502900
C -8.89632100 -1.80763800 0.94932600
H -8.98335400 -0.77307600 1.27543800
H -8.85610900 -2.47260700 1.82017700
C -10.46005000 -3.39161700 -0.08299600
O -9.77682900 -4.37456500 0.22632900
N -10.08022000 -2.11640900 0.15952400
H -10.70577000 -1.34699700 -0.07964200
H -0.30887600 -4.53201800 -1.25547700
H -0.23818200 -2.82925500 -1.78415300
H 0.23818200 2.82925500 1.78415300
H 0.30887600 4.53201800 1.25547700
```

B3LYP

Monomer 12

Zero-point correction= 1.383045 (Hartree/Particle)
Thermal correction to Energy= 1.493658
Thermal correction to Enthalpy= 1.494602
Thermal correction to Gibbs Free Energy= 1.167708
Sum of electronic and zero-point Energies= -5031.438201
Sum of electronic and thermal Energies= -5031.327588
Sum of electronic and thermal Enthalpies= -5031.326644
Sum of electronic and thermal Free Energies= -5031.653538
E(RB3LYP)= -5032.821246

```
C -0.59627900 -14.29146600 0.00000000
C 0.38834800 -13.11477800 0.00000000
C 0.60202500 -10.67446700 0.00000000
H 1.25539100 -10.62636800 0.88095700
H 1.25539100 -10.62636800 -0.88095700
C -0.38294200 -9.49805500 0.00000000
C -0.59708100 -7.05773900 0.00000000
H -1.25044900 -7.00975400 -0.88097400
O 1.60538900 -13.30135000 0.00000000
N -0.17666300 -11.89176700 0.00000000
H -1.18275800 -11.74478900 0.00000000
O -1.59992300 -9.68504500 0.00000000
N 0.18175200 -8.27492900 0.00000000
H 1.18782500 -8.12772800 0.00000000
H -1.25044900 -7.00975400 0.88097400
C 0.38779900 -5.88117100 0.00000000
O 1.60479900 -6.06808600 0.00000000
N -0.17692800 -4.65808200 0.00000000
C 0.60191700 -3.44085700 0.00000000
H -1.18300000 -4.51086400 0.00000000
H 1.25526400 -3.39282600 0.88096700
H 1.25526400 -3.39282600 -0.88096700
C -0.38303400 -2.26441100 0.00000000
O -1.60000900 -2.45147100 0.00000000
N 0.18166500 -1.04131700 0.00000000
C -0.59709800 0.17593000 0.00000000
H 1.18774600 -0.89411800 0.00000000
H -1.25045300 0.22395900 -0.88098000
H -1.25045300 0.22395900 0.88098000
C 0.38791900 1.35240400 0.00000000
C 0.60232500 3.79269300 0.00000000
H 1.25567000 3.84065200 0.88097000
H 1.25567000 3.84065200 -0.88097000
C -0.38252700 4.96922200 0.00000000
```

C -0.59643400 7.40956600 0.00000000
H -1.24978700 7.45763100 -0.88097900
O 1.60489400 1.16531400 0.00000000
N -0.17665500 2.57554800 0.00000000
H -1.18271200 2.72289500 0.00000000
O -1.59951300 4.78225200 0.00000000
N 0.18226400 6.19227500 0.00000000
H 1.18835500 6.33939600 0.00000000
H -1.24978700 7.45763100 0.88097900
C 0.38863600 8.58598600 0.00000000
O 1.60560000 8.39886100 0.00000000
N -0.17587100 9.80917400 0.00000000
C 0.60324300 11.02622500 0.00000000
H -1.18191400 9.95657100 0.00000000
H 1.25660000 11.07409700 0.88096900
H 1.25660000 11.07409700 -0.88096900
C -0.38142600 12.20289100 0.00000000
O -1.59843700 12.01616400 0.00000000
N 0.18353800 13.42589600 0.00000000
H 1.18963900 13.57286700 0.00000000
C -0.59512100 14.64320600 0.00000000
C 0.38989300 15.81962600 0.00000000
C 0.60462700 18.25976900 0.00000000
H 1.25801400 18.30751600 0.88096300
H 1.25801400 18.30751600 -0.88096300
C -0.37970700 19.43666500 0.00000000
C -0.59311700 21.87688600 0.00000000
H -1.24651400 21.92493300 -0.88095400
O 1.60686100 15.63264700 0.00000000
N -0.17465100 17.04284900 0.00000000
H -1.18068300 17.19020000 0.00000000
O -1.59676500 19.25044100 0.00000000
N 0.18556800 20.65961300 0.00000000
H 1.19167900 20.80630300 0.00000000
H -1.24651400 21.92493300 0.88095400
C 0.39162300 23.05341900 0.00000000
O 1.60862600 22.86695300 0.00000000
N -0.17314900 24.27667000 0.00000000
C 0.60641900 25.49334400 0.00000000
H -1.17917000 24.42379200 0.00000000
H 1.25987900 25.54085800 0.88094600
H 1.25987900 25.54085800 -0.88094600
C -0.37719900 26.67070000 0.00000000
O -1.59434700 26.48561500 0.00000000
N 0.18874000 27.89356600 0.00000000
C -0.59015000 29.11065200 0.00000000
H 1.19486000 28.03962700 0.00000000
H -1.24364600 29.15853200 -0.88091200
H -1.24364600 29.15853200 0.88091200
C 0.39372700 30.28759900 0.00000000
C 0.60862900 32.72689200 0.00000000
H 1.26227600 32.77378600 0.88090300
H 1.26227600 32.77378600 -0.88090300
C -0.37303000 33.90545700 0.00000000
C -0.58496700 36.34458300 0.00000000
H -1.23876600 36.39191600 -0.88078300
O 1.61085100 30.10266100 0.00000000
N -0.17174700 31.51088900 0.00000000
H -1.17774500 31.65733400 0.00000000
O -1.59037600 33.72354700 0.00000000
N 0.19475000 35.12817200 0.00000000
H 1.20087700 35.27228700 0.00000000
H -1.23876600 36.39191600 0.88078300
C 0.39592000 37.52290800 0.00000000
O 1.61363200 37.34325200 0.00000000
N -0.17148600 38.74636000 0.00000000
C 0.61263000 39.95882800 0.00000000
H -1.17719300 38.89259300 0.00000000
H 1.26680200 40.00107700 0.88126300
H 1.26680200 40.00107700 -0.88126300
C -0.36085200 41.14506600 0.00000000
O -1.57593200 40.96689000 0.00000000
N 0.22966300 42.36600900 0.00000000
C -0.52343000 43.61098600 0.00000000
H 1.23945300 42.41347100 0.00000000
H -0.29728000 44.20556800 0.89203100
H -0.29728000 44.20556800 -0.89203100
H -1.58303200 43.35420000 0.00000000
H -1.24848400 14.69128600 -0.88097200
H -1.24848400 14.69128600 0.88097200
C -0.53122000 -43.25122900 0.00000000
C 0.41652400 -42.05993200 0.00000000
C 0.58909800 -39.61735100 0.00000000
H 1.24658400 -39.56586100 0.87890600

H 1.24658400 -39.56586100 -0.87890600
C -0.38673200 -38.43682700 0.00000000
C -0.58500800 -35.99632300 0.00000000
H -1.23977500 -35.94635800 -0.88029300
O 1.63671400 -42.19356100 0.00000000
N -0.17884100 -40.83720300 0.00000000
H -1.18424200 -40.70966700 0.00000000
O -1.60652800 -38.60279900 0.00000000
N 0.18912600 -37.21486400 0.00000000
H 1.19573800 -37.07822300 0.00000000
H -1.23977500 -35.94635800 0.88029300
C 0.39563700 -34.81787200 0.00000000
O 1.61360400 -34.99518500 0.00000000
N -0.17481600 -33.59538800 0.00000000
C 0.60184500 -32.37759100 0.00000000
H -1.18112500 -33.45328900 0.00000000
H 1.25590400 -32.32875300 0.88063700
H 1.25590400 -32.32875300 -0.88063700
C -0.38089100 -31.20010200 0.00000000
O -1.59843700 -31.38217400 0.00000000
N 0.18653700 -29.97724500 0.00000000
C -0.59159700 -28.75999700 0.00000000
H 1.19271100 -29.83249400 0.00000000
H -1.24532600 -28.71182200 -0.88080100
H -1.24532600 -28.71182200 0.88080100
C 0.39167300 -27.58260700 0.00000000
C 0.60304500 -25.14235500 0.00000000
H 1.25660400 -25.09405200 0.88087000
H 1.25660400 -25.09405200 -0.88087000
C -0.38130100 -23.96565000 0.00000000
C -0.59463900 -21.52545300 0.00000000
H -1.24812100 -21.47749800 -0.88091900
O 1.60899300 -27.76647500 0.00000000
N -0.17501300 -26.35983700 0.00000000
H -1.18118700 -26.21435100 0.00000000
O -1.59843700 -24.15121900 0.00000000
N 0.18413700 -22.74256500 0.00000000
H 1.19022500 -22.59602300 0.00000000
H -1.24812100 -21.47749800 0.88091900
C 0.38958000 -20.34852000 0.00000000
O 1.60670200 -20.53432200 0.00000000
N -0.17591600 -19.12559300 0.00000000
C 0.60256600 -17.90822000 0.00000000
H -1.18203600 -18.97903900 0.00000000
H 1.25598600 -17.86006000 0.88093200
H 1.25598600 -17.86006000 -0.88093200
C -0.38224200 -16.73173200 0.00000000
O -1.59926800 -16.91835400 0.00000000
N 0.18262200 -15.50860400 0.00000000
H 1.18869100 -15.36152700 0.00000000
H -1.58919900 -42.97108400 0.00000000
H -0.32544400 -43.86645900 0.88154900
H -1.24967600 -14.24350500 0.88094900
H -1.24967600 -14.24350500 -0.88094900
H -0.32544400 -43.86645900 -0.88154900

B3LYP

Dimer 12

Zero-point correction= 2.816177 (Hartree/Particle)
Thermal correction to Energy= 3.017681
Thermal correction to Enthalpy= 3.018625
Thermal correction to Gibbs Free Energy= 2.525200
Sum of electronic and zero-point Energies= -10063.025918
Sum of electronic and thermal Energies= -10062.824414
Sum of electronic and thermal Enthalpies= -10062.823470
Sum of electronic and thermal Free Energies= -10063.316895
E(RB3LYP)= -10065.842094

C -22.90601900 3.20114500 -1.73498200
C -24.23750300 2.58116800 -2.19231100
H -22.83833400 4.18621800 -2.19700600
H -22.91165500 3.31098400 -0.64375400
C -25.77166000 0.68356900 -1.95150700
H -25.78953200 0.60519900 -3.04489400
H -25.67899100 -0.30671100 -1.50926700
C -27.09440100 1.28988000 -1.46230900

C -28.72182700 3.09586200 -1.75854700
H -28.66440600 4.10181300 -2.17518900
O -24.91378800 3.10161500 -3.08641600
N -24.60751200 1.45540300 -1.54017400
H -23.98687100 1.04378100 -0.84336000
O -27.71672900 0.82937600 -0.49809200
N -27.53522100 2.35555600 -2.16860000
H -26.88020800 2.76633200 -2.83388700
H -28.74741100 3.15415700 -0.66424100
C -23.38158000 -3.09272700 1.87160400
H -23.46762200 -4.05018500 2.38566600
H -23.39236800 -3.26008900 0.78777200
C -26.20659300 -0.52825100 1.95672300
H -26.15064500 -0.36755600 3.03969300
H -26.27839700 0.42808900 1.44252200
C -29.18335500 -2.92249400 2.04285500
H -29.31077300 -3.81756400 2.65164800
N -24.52331700 -2.26475200 2.24048900
H -25.18291900 -2.59169000 2.94761300
C -24.92196500 -1.21991000 1.48135600
O -24.29371100 -0.83719400 0.48533600
N -27.41090300 -1.28323600 1.64421000
H -28.01378300 -0.92728900 0.90191600
O -27.13389500 -2.79498400 3.32647700
C -27.81256300 -2.32567700 2.40542800
C -30.02999000 2.47329300 -2.27523300
O -30.65533700 2.99546400 -3.20570300
N -30.44054400 1.35024200 -1.64183800
C -31.55942900 0.55040800 -2.13213100
H -29.85470900 0.92836600 -0.92060500
H -31.50001300 0.48088600 -3.22525100
H -31.46221200 -0.44355600 -1.69849800
C -32.94911000 1.09323400 -1.74514500
O -33.70639500 0.47337300 -0.98589500
N -33.27041100 2.26718900 -2.32598500
H -32.53826400 2.71200600 -2.88171300
H -29.18588600 -3.20198300 0.98200500
N -30.29911000 -2.01986800 2.30660500
H -31.00613800 -2.27817500 2.99568300
C -30.65060700 -1.05060900 1.43269900
O -29.99778600 -0.79825800 0.41125300
C -31.92865700 -0.28529700 1.79285600
H -31.91155300 0.66429000 1.25746100
H -31.96161000 -0.09748200 2.87105900
C -33.60575900 -2.02632100 2.14627200
O -33.02937100 -2.42231700 3.16760200
N -33.12648900 -1.00485000 1.39596200
H -33.55053400 -0.71002200 0.51624900
C -12.66573000 2.59109000 -2.17182500
C -14.22727300 0.71708400 -1.91253400
H -14.25398000 0.63287300 -3.00535700
H -14.14621500 -0.27184100 -1.46551500
C -15.53714400 1.34824100 -1.41939800
C -17.11101100 3.20973500 -1.71716200
H -17.03858100 4.19553000 -2.17693500
O -13.33789300 3.11687600 -3.06633900
N -13.04877900 1.47364200 -1.51333100
H -12.42923900 1.05491700 -0.81984100
O -16.16825900 0.89496200 -0.45564100
N -15.95165200 2.42480100 -2.12388900
H -15.28414500 2.81480200 -2.79027500
H -17.11062400 3.31671000 -0.62563800
C -14.68856500 -0.65287500 1.93490300
H -14.65888300 -0.56171400 3.02711000
H -14.77177200 0.33308100 1.48182200
C -17.59067900 -3.13432300 1.77067600
H -17.66577500 -4.11390200 2.24316200
C -13.37927100 -1.28590500 1.44268500
O -12.74817800 -0.83547700 0.47753600
N -15.86770500 -1.41290200 1.54410300
H -16.48919100 -1.00013700 0.84868900
O -15.57341000 -3.04547200 3.10736300
C -16.24889100 -2.52554700 2.21184900
C -18.44919400 2.60113300 -2.16953900
O -19.12119500 3.12524700 -3.06504300
N -18.83101300 1.48328600 -1.51089900
C -20.00675500 0.72393700 -1.91268100
H -18.21045300 1.06506000 -0.81801800
H -20.03029700 0.63860300 -3.00549700
H -19.92466200 -0.26441600 -1.46451000
C -21.31964700 1.35191900 -1.42353000
O -21.94925600 0.89979700 -0.45830400
N -21.73920200 2.42288700 -2.13369700
H -21.07249500 2.81418900 -2.79992000

H -17.59384000 -3.25531700 0.68064600
N -18.74608000 -2.33972200 2.16900100
H -19.40864100 -2.71236200 2.85040400
C -19.15868800 -1.27165400 1.45084800
O -18.52909600 -0.83494500 0.47829900
C -20.46253900 -0.62643700 1.94127100
H -20.54850900 0.34976700 1.46810600
H -20.42151700 -0.51203700 3.03095800
C -22.02864600 -2.48467200 2.27921100
O -21.34546500 -2.98798200 3.17844000
N -21.64773500 -1.39154300 1.57976100
H -22.27321900 -0.99541400 0.87806600
C 23.38158000 3.09272700 -1.87160400
C 22.02864600 2.48467200 -2.27921100
H 23.46762200 4.05018500 -2.38566600
H 23.39236800 3.26008900 -0.78777200
C 20.46253900 0.62643700 -1.94127100
H 20.42151700 0.51203700 -3.03095800
H 20.54850900 -0.34976700 -1.46810600
C 19.15868800 1.27165400 -1.45084800
C 17.59067900 3.13432300 -1.77067600
H 17.66577500 4.11390200 -2.24316200
O 21.34546500 2.98798200 -3.17844000
N 21.64773500 1.39154300 -1.57976100
H 22.27321900 0.99541400 -0.87806600
O 18.52909600 0.83494500 -0.47829900
N 18.74608000 2.33972200 -2.16900100
H 19.40864100 2.71236200 -2.85040400
H 17.59384000 3.25531700 -0.68064600
C 22.90601900 -3.20114500 1.73498200
H 22.83833400 -4.18621800 2.19700600
H 22.91165500 -3.31098400 0.64375400
C 20.00675500 -0.72393700 1.91268100
H 20.03029700 -0.63860300 3.00549700
H 19.92466200 0.26441600 1.46451000
C 17.11101100 -3.20973500 1.71716200
H 17.03858100 -4.19553000 2.17693500
N 21.73920200 -2.42288700 2.13369700
H 21.07249500 -2.81418900 2.79992000
C 21.31964700 -1.35191900 1.42353000
O 21.94925600 -0.89979700 0.45830400
N 18.83101300 -1.48328600 1.51089900
H 18.21045300 -1.06506000 0.81801800
O 19.12119500 -3.12524700 3.06504300
C 18.44919400 -2.60113300 2.16953900
C 16.24889100 2.52554700 -2.21184900
O 15.57341000 3.04547200 -3.10736300
N 15.86770500 1.41290200 -1.54410300
C 14.68856500 0.65287500 -1.93490300
H 16.48919100 1.00013700 -0.84868900
H 14.65888300 0.56171400 -3.02711000
H 14.77177200 -0.33308100 -1.48182200
C 13.37927100 1.28590500 -1.44268500
O 12.74817800 0.83547700 -0.47753600
N 12.96480200 2.36071500 -2.14982000
C 11.80668100 3.14768200 -1.74388500
H 13.63150200 2.74773900 -2.81891100
H 17.11062400 -3.31671000 0.62563800
N 15.95165200 -2.42480100 2.12388900
H 15.28414500 -2.81480200 2.79027500
C 15.53714400 -1.34824100 1.41939800
O 16.16825900 -0.89496200 0.45564100
C 14.22727300 -0.71708400 1.91253400
H 14.14621500 0.27184100 1.46551500
H 14.25398000 -0.63287300 3.00535700
C 12.66573000 -2.59109000 2.17182500
O 13.33789300 -3.11687600 3.06633900
C 11.32636900 -3.19786400 1.72056000
N 13.04877900 -1.47364200 1.51333100
H 12.42923900 -1.05491700 0.81984100
C 33.60575900 2.02632100 -2.14627200
C 31.92865700 0.28529700 -1.79285600
H 31.96161000 0.09748200 -2.87105900
H 31.91155300 -0.66429000 -1.25746100
C 30.65060700 1.05060900 -1.43269900
C 29.18335500 2.92249400 -2.04285500
H 29.31077300 3.81756400 -2.65164800
O 33.02937100 2.42231700 -3.16760200
N 33.12648900 1.00485000 -1.39596200
H 33.55053400 0.71002200 -0.51624900
O 29.99778600 0.79825800 -0.41125300
N 30.29911000 2.01986800 -2.30660500
H 31.00613800 2.27817500 -2.99568300
H 29.18588600 3.20198300 -0.98200500

C 31.55942900 -0.55040800 2.13213100
H 31.50001300 -0.48088600 3.22525100
H 31.46221200 0.44355600 1.69849800
C 28.72182700 -3.09586200 1.75854700
H 28.66440600 -4.10181300 2.17518900
N 33.27041100 -2.26718900 2.32598500
H 32.53826400 -2.71200600 2.88171300
C 32.94911000 -1.09323400 1.74514500
O 33.70639500 -0.47337300 0.98589500
N 30.44054400 -1.35024200 1.64183800
H 29.85470900 -0.92836600 0.92060500
O 30.65533700 -2.99546400 3.20570300
C 30.02999000 -2.47329300 2.27523300
C 27.81256300 2.32567700 -2.40542800
O 27.13389500 2.79498400 -3.32647700
N 27.41090300 1.28323600 -1.64421000
C 26.20659300 0.52825100 -1.95672300
H 28.01378300 0.92728900 -0.90191600
H 26.15064500 0.36755600 -3.03969300
H 26.27839700 -0.42808900 -1.44252200
C 24.92196500 1.21991000 -1.48135600
O 24.29371100 0.83719400 -0.48533600
N 24.52331700 2.26475200 -2.24048900
H 25.18291900 2.59169000 -2.94761300
H 28.74741100 -3.15415700 0.66424100
N 27.53522100 -2.35555600 2.16860000
H 26.88020800 -2.76633200 2.83388700
C 27.09440100 -1.28988000 1.46230900
O 27.71672900 -0.82937600 0.49809200
C 25.77166000 -0.68356900 1.95150700
H 25.67899100 0.30671100 1.50926700
H 25.78953200 -0.60519900 3.04489400
C 24.23750300 -2.58116800 2.19231100
O 24.91378800 -3.10161500 3.08641600
N 24.60751200 -1.45540300 1.54017400
H 23.98687100 -1.04378100 0.84336000
C 0.24031400 3.17226500 -1.73125800
C -1.09902600 2.56524400 -2.18223300
H 0.31319500 4.15793100 -2.19123700
H 0.24166400 3.27943900 -0.63974400
C -2.66049100 0.69127200 -1.92209600
H -2.68723100 0.60660100 -3.01488400
H -2.57939300 -0.29745300 -1.47464500
C -3.97030400 1.32268200 -1.42918700
C -5.54307700 3.18491000 -1.72630100
H -5.47023100 4.17063400 -2.18616000
O -1.77126900 3.09059100 -3.07696100
N -1.48204800 1.44808300 -1.52323500
H -0.86267200 1.02985800 -0.82928700
O -4.60155800 0.86996100 -0.46528000
N -4.38470400 2.39909800 -2.13396900
H -3.71699200 2.78899300 -2.80026400
H -5.54168500 3.29195000 -0.63477500
C -0.24031400 -3.17226500 1.73125800
H -0.31319500 -4.15793100 2.19123700
H -0.24166400 -3.27943900 0.63974400
C -3.12286800 -0.67857300 1.92666400
H -3.09613000 -0.59371300 3.01943900
H -3.20401400 0.31006900 1.47904100
C -6.02355600 -3.15972100 1.73629500
H -6.09644000 -4.14527800 2.19650800
N -1.39867500 -2.38637200 2.13879600
H -2.06649400 -2.77622500 2.80502400
C -1.81301200 -1.31000400 1.43389800
O -1.18167100 -0.85734900 0.47000900
N -4.30127200 -1.43551000 1.52793700
H -4.92075800 -1.01739400 0.83401500
O -4.01184600 -3.07784400 3.08180800
C -4.68420300 -2.55261300 2.18708900
C -6.88243400 2.57794300 -2.17731300
O -7.55466200 3.10341000 -3.07197900
N -7.26545600 1.46071000 -1.51844300
C -8.44400000 0.70402700 -1.91726800
H -6.64613100 1.04243400 -0.82448500
H -8.47088300 0.61954400 -3.01006500
H -8.36289800 -0.28477900 -1.46999800
C -9.75371300 1.33543000 -1.42406900
O -10.38493400 0.88246300 -0.46025800
N -10.16802900 2.41212100 -2.12846600
C -11.32636900 3.19786400 -1.72056000
H -9.50043400 2.80206100 -2.79483800
H -6.02490300 -3.26716100 0.64480500
N -7.18190000 -2.37372600 2.14363800
H -7.84964500 -2.76331700 2.81012100

C -7.59628900 -1.29755500 1.43847300
O -6.96491700 -0.84506800 0.47451300
C -8.90617900 -0.66607500 1.93110200
H -8.98768800 0.32221500 1.48278200
H -8.87912300 -0.58041300 3.02380900
C -10.46714100 -2.54017000 2.19352200
O -9.79433000 -3.06465000 3.08835800
C -11.80668100 -3.14768200 1.74388500
N -10.08457200 -1.42355000 1.53333000
H -10.70432900 -1.00623000 0.83915400
C 10.46714100 2.54017000 -2.19352200
C 8.90617900 0.66607500 -1.93110200
H 8.87912300 0.58041300 -3.02380900
H 8.98768800 -0.32221500 -1.48278200
C 7.59628900 1.29755500 -1.43847300
C 6.02355600 3.15972100 -1.73629500
H 6.09644000 4.14527800 -2.19650800
O 9.79433000 3.06465000 -3.08835800
N 10.08457200 1.42355000 -1.53333000
H 10.70432900 1.00623000 -0.83915400
O 6.96491700 0.84506800 -0.47451300
N 7.18190000 2.37372600 -2.14363800
H 7.84964500 2.76331700 -2.81012100
H 6.02490300 3.26716100 -0.64480500
C 8.44400000 -0.70402700 1.91726800
H 8.47088300 -0.61954400 3.01006500
H 8.36289800 0.28477900 1.46999800
C 5.54307700 -3.18491000 1.72630100
H 5.47023100 -4.17063400 2.18616000
N 10.16802900 -2.41212100 2.12846600
H 9.50043400 -2.80206100 2.79483800
C 9.75371300 -1.33543000 1.42406900
O 10.38493400 -0.88246300 0.46025800
N 7.26545600 -1.46071000 1.51844300
H 6.64613100 -1.04243400 0.82448500
O 7.55466200 -3.10341000 3.07197900
C 6.88243400 -2.57794300 2.17731300
C 4.68420300 2.55261300 -2.18708900
O 4.01184600 3.07784400 -3.08180800
N 4.30127200 1.43551000 -1.52793700
C 3.12286800 0.67857300 -1.92666400
H 4.92075800 1.01739400 -0.83401500
H 3.09613000 0.59371300 -3.01943900
H 3.20401400 -0.31006900 -1.47904100
C 1.81301200 1.31000400 -1.43389800
O 1.18167100 0.85734900 -0.47000900
N 1.39867500 2.38637200 -2.13879600
H 2.06649400 2.77622500 -2.80502400
H 5.54168500 -3.29195000 0.63477500
N 4.38470400 -2.39909800 2.13396900
H 3.71699200 -2.78899300 2.80026400
C 3.97030400 -1.32268200 1.42918700
O 4.60155800 -0.86996100 0.46528000
C 2.66049100 -0.69127200 1.92209600
H 2.57939300 0.29745300 1.47464500
H 2.68723100 -0.60660100 3.01488400
C 1.09902600 -2.56524400 2.18223300
O 1.77126900 -3.09059100 3.07696100
N 1.48204800 -1.44808300 1.52323500
H 0.86267200 -1.02985800 0.82928700
H 11.25345600 -4.18373900 2.18008600
H 11.32497700 -3.30453000 0.62899900
H 11.80811200 3.25694800 -0.65258400
H 11.87977400 4.13245900 -2.20574400
N -12.96480200 -2.36071500 2.14982000
H -13.63150200 -2.74773900 2.81891100
H -11.87977400 -4.13245900 2.20574400
H -11.80811200 -3.25694800 0.65258400
H -11.32497700 3.30453000 -0.62899900
H -11.25345600 4.18373900 -2.18008600
C 34.51594600 -2.96649400 2.06627400
C 34.90352800 2.65180800 -1.67120800
C -34.90352800 -2.65180800 1.67120800
C -34.51594600 2.96649400 -2.06627400
H -34.34009800 3.89849400 -1.51512300
H -35.02349000 3.20789300 -3.00655700
H -35.15422800 2.31400900 -1.46940100
H 35.02349000 -3.20789300 3.00655700
H 34.34009800 -3.89849400 1.51512300
H 35.15422800 -2.31400900 1.46940100
H 35.65966700 2.52855300 -2.45322000
H 34.75184200 3.72662800 -1.53227800
H 35.27704800 2.21470800 -0.74109000
H -34.75184200 -3.72662800 1.53227800

H -35.65966700 -2.52855300 2.45322000
H -35.27704800 -2.21470800 0.74109000

B3LYP

Monomer 16

Zero-point correction= 1.828415 (Hartree/Particle)
Thermal correction to Energy= 1.975238
Thermal correction to Enthalpy= 1.976182
Thermal correction to Gibbs Free Energy= 1.550043
Sum of electronic and zero-point Energies= -6695.097156
Sum of electronic and thermal Energies= -6694.950333
Sum of electronic and thermal Enthalpies= -6694.949389
Sum of electronic and thermal Free Energies= -6695.375528
E(RB3LYP)= -6696.925571

```
C -0.55416000 0.18034100 0.00000000
C 0.43799000 1.35077200 0.00000000
C 0.66632400 3.79007900 0.00000000
H 1.31990100 3.83447800 0.88098900
H 1.31990100 3.83447800 -0.88098900
C -0.31236000 4.97188300 0.00000000
C -0.51424600 7.41316100 0.00000000
H -1.16735700 7.46435000 -0.88097700
O 1.65380000 1.15615700 0.00000000
N -0.11933700 2.57719600 0.00000000
H -1.12454500 2.73038500 0.00000000
O -1.53031800 4.79111800 0.00000000
N 0.25854400 6.19205200 0.00000000
H 1.26534800 6.33429800 0.00000000
H -1.16735700 7.46435000 0.88097700
C 0.47650700 8.58479500 0.00000000
O 1.69254100 8.39166200 0.00000000
N -0.08232200 9.81054700 0.00000000
C 0.70184000 11.02437500 0.00000000
H -1.08772400 9.96246000 0.00000000
H 1.35533700 11.06958700 0.88098000
H 1.35533700 11.06958700 -0.88098000
C -0.27827700 12.20498700 0.00000000
O -1.49600500 12.02276900 0.00000000
N 0.29122200 13.42581700 0.00000000
C -0.48289700 14.64607000 0.00000000
H 1.29786200 13.56918500 0.00000000
H -1.13607700 14.69656700 -0.88097500
H -1.13607700 14.69656700 0.88097500
C 0.50658100 15.81877900 0.00000000
C 0.72948300 18.25852600 0.00000000
H 1.38295600 18.30437800 0.88098800
H 1.38295600 18.30437800 -0.88098800
C -0.25176000 19.43816200 0.00000000
C -0.45891400 21.87897700 0.00000000
H -1.11213700 21.92874700 -0.88096900
O 1.72282400 15.62699400 0.00000000
N -0.05351900 17.04395400 0.00000000
H -1.05906900 17.19484200 0.00000000
O -1.46930500 19.25477300 0.00000000
N 0.31653300 20.65957300 0.00000000
H 1.32302300 20.80395000 0.00000000
H -1.11213700 21.92874700 0.88096900
C 0.52917100 23.05284800 0.00000000
O 1.74565300 22.86261000 0.00000000
N -0.03246700 24.27734800 0.00000000
C 0.74903700 25.49288200 0.00000000
H -1.03820400 24.42694500 0.00000000
H 1.40247000 25.53954300 0.88098800
H 1.40247000 25.53954300 -0.88098800
C -0.23365500 26.67129100 0.00000000
O -1.45097400 26.48646100 0.00000000
N 0.33302300 27.89349700 0.00000000
H 1.33930400 28.03924100 0.00000000
C -0.44432300 29.11168600 0.00000000
C 0.54173500 30.28721400 0.00000000
C 0.75739200 32.72758600 0.00000000
H 1.41076500 32.77532400 0.88098000
H 1.41076500 32.77532400 -0.88098000
C -0.22712400 33.90440300 0.00000000
C -0.44213800 36.34426200 0.00000000
```

H -1.09560000 36.39164700 -0.88093700
O 1.75854800 30.09922500 0.00000000
N -0.02208700 31.51077600 0.00000000
H -1.02808000 31.65852700 0.00000000
O -1.44415300 33.71783200 0.00000000
N 0.33758400 35.12761200 0.00000000
H 1.34359500 35.27498100 0.00000000
H -1.09560000 36.39164700 0.88093700
C 0.54132800 37.52185900 0.00000000
O 1.75854800 37.33682700 0.00000000
N -0.02514400 38.74433900 0.00000000
C 0.75221300 39.96244400 0.00000000
H -1.03141900 38.88985200 0.00000000
H 1.40556100 40.01126000 0.88096000
H 1.40556100 40.01126000 -0.88096000
C -0.23389300 41.13777500 0.00000000
O -1.45064800 40.94996800 0.00000000
N 0.32910300 42.36199800 0.00000000
C -0.45317100 43.57694200 0.00000000
H 1.33483800 42.51067900 0.00000000
H -1.10683100 43.62288600 -0.88089500
H -1.10683100 43.62288600 0.88089500
C 0.52713400 44.75686800 0.00000000
C 0.73356800 47.19717300 0.00000000
H 1.38701600 47.24654900 0.88090800
H 1.38701600 47.24654900 -0.88090800
C -0.25267700 48.37195300 0.00000000
C -0.47489000 50.81002300 0.00000000
H -1.12891800 50.85445500 -0.88077700
O 1.74483200 44.57573700 0.00000000
N -0.04237200 45.97830900 0.00000000
H -1.04888700 46.12126000 0.00000000
O -1.46930400 48.18521000 0.00000000
N 0.31008700 49.59699300 0.00000000
H 1.31560600 49.74534000 0.00000000
H -1.12891800 50.85445500 0.88077700
C 0.50070000 51.99275900 0.00000000
O 1.71922700 51.81862700 0.00000000
N -0.07236000 53.21359900 0.00000000
C 0.70599300 54.42981700 0.00000000
H -1.07875300 53.35513400 0.00000000
H 1.35994200 54.47522700 0.88125800
H 1.35994200 54.47522700 -0.88125800
C -0.27326700 55.61128600 0.00000000
O -1.48748100 55.42709700 0.00000000
N 0.31123500 56.83514100 0.00000000
C -0.44807100 58.07640600 0.00000000
H 1.32079200 56.88760900 0.00000000
H -1.50641700 57.81444900 0.00000000
H -0.22489800 58.67208200 0.89200100
H -0.22489800 58.67208200 -0.89200100
H -1.09764300 29.16040200 -0.88095800
H -1.09764300 29.16040200 0.88095800
C -0.71889400 -57.71756900 0.00000000
C 0.22857500 -56.52602600 0.00000000
H -0.51295300 -58.33272700 0.88151700
H -1.77696800 -57.43781800 0.00000000
H -0.51295300 -58.33272700 -0.88151700
C 0.40068200 -54.08342600 0.00000000
H 1.05815700 -54.03183300 0.87889300
H 1.05815700 -54.03183300 -0.87889300
C -0.57532500 -52.90307900 0.00000000
C -0.77363800 -50.46249800 0.00000000
H -1.42840300 -50.41250000 -0.88030100
O 1.44880600 -56.65937300 0.00000000
N -0.36705600 -55.30342100 0.00000000
H -1.37247700 -55.17602800 0.00000000
O -1.79509300 -53.06924100 0.00000000
N 0.00043600 -51.68107200 0.00000000
H 1.00705000 -51.54441800 0.00000000
H -1.42840300 -50.41250000 0.88030100
C 0.20715100 -49.28411900 0.00000000
O 1.42508900 -49.46163600 0.00000000
N -0.36299700 -48.06149800 0.00000000
C 0.41413600 -46.84399000 0.00000000
H -1.36925300 -47.91907400 0.00000000
H 1.06821600 -46.79544300 0.88062900
H 1.06821600 -46.79544300 -0.88062900
C -0.56804900 -45.66605500 0.00000000
O -1.78566900 -45.84753700 0.00000000
N 0.00014400 -44.44355600 0.00000000
C -0.77689700 -43.22561500 0.00000000
H 1.00643400 -44.29956500 0.00000000
H -1.43056600 -43.17678500 -0.88081400

H -1.43056600 -43.17678500 0.88081400
C 0.20761200 -42.04923200 0.00000000
C 0.42238200 -39.60945000 0.00000000
H 1.07602700 -39.56224000 0.88086000
H 1.07602700 -39.56224000 -0.88086000
C -0.55998100 -38.43108700 0.00000000
C -0.76808600 -35.99015100 0.00000000
H -1.42141900 -35.94052800 -0.88093700
O 1.42472300 -42.23451000 0.00000000
N -0.35753000 -40.82575800 0.00000000
H -1.36348500 -40.67886000 0.00000000
O -1.77741800 -38.61453500 0.00000000
N 0.00781800 -37.20910200 0.00000000
H 1.01422800 -37.06470000 0.00000000
H -1.42141900 -35.94052800 0.88093700
C 0.21921500 -34.81576700 0.00000000
O 1.43582900 -35.00495300 0.00000000
N -0.34269300 -33.59120000 0.00000000
C 0.43982400 -32.37640200 0.00000000
H -1.34833800 -33.44149300 0.00000000
H 1.09341400 -32.33054100 0.88092600
H 1.09341400 -32.33054100 -0.88092600
C -0.54082000 -31.19643500 0.00000000
O -1.75849300 -31.37865700 0.00000000
N 0.02877700 -29.97550400 0.00000000
H 1.03546100 -29.83257400 0.00000000
C -0.74473100 -28.75492900 0.00000000
C 0.24545300 -27.58281400 0.00000000
C 0.47189700 -25.14388800 0.00000000
H 1.12553000 -25.09946200 0.88095200
H 1.12553000 -25.09946200 -0.88095200
C -0.50642600 -23.96192300 0.00000000
C -0.70564000 -21.52002700 0.00000000
H -1.35865100 -21.46779800 -0.88098500
O 1.46159100 -27.77530400 0.00000000
N -0.31336900 -26.35697700 0.00000000
H -1.31867100 -26.20470400 0.00000000
O -1.72444400 -24.14200400 0.00000000
N 0.06547200 -22.74215500 0.00000000
H 1.07243700 -22.60105600 0.00000000
H -1.35865100 -21.46779800 0.88098500
C 0.28695900 -20.34990300 0.00000000
O 1.50269400 -20.54498400 0.00000000
N -0.26947400 -19.12304600 0.00000000
C 0.51776500 -17.91121800 0.00000000
H -1.27451000 -18.96889100 0.00000000
H 1.17143900 -17.86779000 0.88096800
H 1.17143900 -17.86779000 -0.88096800
C -0.45891400 -16.72784700 0.00000000
O -1.67717300 -16.90640400 0.00000000
N 0.11443500 -15.50880900 0.00000000
C -0.65541300 -14.28586800 0.00000000
H 1.12155300 -15.36878400 0.00000000
H -1.30836200 -14.23302200 -0.88098400
H -1.30836200 -14.23302200 0.88098400
C 0.33832300 -13.11670900 0.00000000
C 0.57059700 -10.67796200 0.00000000
H 1.22429300 -10.63472100 0.88098000
H 1.22429300 -10.63472100 -0.88098000
C -0.40581700 -9.49433700 0.00000000
C -0.60252000 -7.05252700 0.00000000
H -1.25547700 -6.99985600 -0.88097500
O 1.55386200 -13.31296300 0.00000000
N -0.21715200 -11.88945000 0.00000000
H -1.22210400 -11.73464600 0.00000000
O -1.62411400 -9.67276300 0.00000000
N 0.16755700 -8.27534100 0.00000000
H 1.17466000 -8.13520400 0.00000000
H -1.25547700 -6.99985600 0.88097500
C 0.39094700 -5.88315800 0.00000000
O 1.60653100 -6.07913900 0.00000000
N -0.16497200 -4.65610800 0.00000000
C 0.62209000 -3.44414000 0.00000000
H -1.17000500 -4.50175100 0.00000000
H 1.27569900 -3.40048700 0.88100200
H 1.27569900 -3.40048700 -0.88100200
C -0.35517900 -2.26117800 0.00000000
O -1.57335500 -2.44052700 0.00000000
N 0.21717400 -1.04171000 0.00000000
H 1.22413800 -0.90061000 0.00000000
H -1.39787400 -28.70396500 -0.88097500
H -1.39787400 -28.70396500 0.88097500
H -1.20723200 0.23227200 0.88095800
H -1.20723200 0.23227200 -0.88095800

B3LYP

Dimer 16

Zero-point correction= 3.723670 (Hartree/Particle)

Thermal correction to Energy= 3.990230

Thermal correction to Enthalpy= 3.991174

Thermal correction to Gibbs Free Energy= 3.348311

Sum of electronic and zero-point Energies= -13390.386409

Sum of electronic and thermal Energies= -13390.119849

Sum of electronic and thermal Enthalpies= -13390.118905

Sum of electronic and thermal Free Energies= -13390.761768

E(RB3LYP)= -13394.110079

```
C -11.32613800 3.51851400 0.89298100
C -12.66627100 3.37218400 0.15249900
H -11.25338600 4.55723000 1.21578000
H -11.32340600 2.86340900 1.77259300
C -14.22815000 1.80981300 -0.91394900
H -14.25619100 2.48228700 -1.77943100
H -14.14686300 0.77729200 -1.24809000
C -15.53719200 1.94469800 -0.12310300
C -17.10950700 3.52156800 0.91183900
H -17.03677100 4.56019800 1.23491700
O -13.33929800 4.36248900 -0.15532900
N -13.04899100 2.10209900 -0.11100500
H -12.42890700 1.32619900 0.12042500
O -16.16771200 0.96093900 0.28580200
N -15.95179300 3.21505200 0.08042300
H -15.28471800 3.95212200 -0.15096600
H -17.10704300 2.86624000 1.79128200
C -11.80662700 -3.50592600 -0.81332900
H -11.87932000 -4.54464900 -1.13611700
H -11.80927400 -2.85084800 -1.69296700
C -14.68811800 -1.79406200 1.01213300
H -14.66031100 -2.46657400 1.87759600
H -14.76945700 -0.76155100 1.34628900
C -17.58963200 -3.50260600 -0.79575500
H -17.66229000 -4.54131800 -1.11859400
N -12.96424900 -3.19933400 0.01818500
H -13.63157200 -3.93631600 0.24921200
C -13.37887900 -1.92896300 0.22160700
O -12.74822300 -0.94521800 -0.18715500
N -15.86706900 -2.08625800 0.20884900
H -16.48729800 -1.31040100 -0.02239600
O -15.57666500 -4.34663600 0.25292900
C -16.24971500 -3.35632900 -0.05487800
C -18.44944800 3.37549700 0.17095800
O -19.12235000 4.36592400 -0.13674800
N -18.83212600 2.10550500 -0.09304300
C -20.01113400 1.81347100 -0.89630900
H -18.21218500 1.32949800 0.13840500
H -20.03901400 2.48624300 -1.76156400
H -19.92976600 0.78106800 -1.23079400
C -21.32033400 1.94805700 -0.10567100
O -21.95098900 0.96412100 0.30261000
N -21.73486900 3.21833300 0.09842100
C -22.89278900 3.52454700 0.92967100
H -21.06779100 3.95549400 -0.13264500
H -17.59198200 -2.84750400 -1.67537500
N -18.74748500 -3.19596500 0.03540800
H -19.41480300 -3.93296400 0.26645700
C -19.16221700 -1.92559600 0.23861900
O -18.53140200 -0.94185600 -0.16993700
C -20.47175500 -1.79070000 1.02865700
H -20.55354600 -0.75805400 1.36226800
H -20.44395500 -2.46277500 1.89446300
C -22.03264000 -3.35408200 -0.03778700
O -21.35928100 -4.34399000 0.27066800
C -23.37247200 -3.50124600 -0.77860800
N -21.65041700 -2.08372700 0.22523300
H -22.27081600 -1.30822200 -0.00677800
C -1.09991900 3.36597900 0.11259000
C -2.66207500 1.80322200 -0.95292500
H -2.69045900 2.47546100 -1.81858300
H -2.58081300 0.77061800 -1.28681300
C -3.97086800 1.93820800 -0.16168900
```

C -5.54289000 3.51520800 0.87351700
H -5.47008000 4.55389700 1.19639300
O -1.77314400 4.35615500 -0.19520100
N -1.48267100 2.09579300 -0.15043100
H -0.86230100 1.32004200 0.08075100
O -4.60125500 0.95450000 0.24756000
N -4.38543300 3.20858100 0.04179200
H -3.71850200 3.94564500 -0.19003900
H -5.54010800 2.86003700 1.75307500
C -3.12119300 -1.80044100 0.97388300
H -3.09300700 -2.47292100 1.83935500
H -3.20236000 -0.76791800 1.30805000
C -6.02355300 -3.50907800 -0.83254200
H -6.09633700 -4.54778100 -1.15537600
C -1.81234900 -1.93541000 0.18272100
O -1.18197900 -0.95170800 -0.22657600
N -4.30052500 -2.09266900 0.17117000
H -4.92069700 -1.31676600 -0.06003200
O -4.01025100 -4.35305700 0.21552800
C -4.68333300 -3.36276300 -0.09221500
C -6.88308400 3.36892900 0.13313800
O -7.55615300 4.35923600 -0.17459100
N -7.26581400 2.09884900 -0.13039400
C -8.44510700 1.80656800 -0.93314300
H -6.64577500 1.32291900 0.10105200
H -8.47326500 2.47901100 -1.79864400
H -8.36389300 0.77403300 -1.26726200
C -9.75402100 1.94151600 -0.14209300
O -10.38444600 0.95779200 0.26705200
N -10.16862600 3.21189200 0.06131900
H -9.50152100 3.94891800 -0.17014100
H -6.02635100 -2.85394100 -1.71213100
N -7.18101100 -3.20248000 -0.00079900
H -7.84818600 -3.93948900 0.23054400
C -7.59561400 -1.93210600 0.20263900
O -6.96511200 -0.94838000 -0.20639700
C -8.90460900 -1.79716800 0.99356600
H -8.98586300 -0.76462700 1.32765400
H -8.87651000 -2.46959300 1.85908600
C -10.46651000 -3.35956500 -0.07281900
O -9.79339500 -4.34984600 0.23491700
N -10.08381400 -2.08947300 0.19070300
H -10.70403700 -1.31362300 -0.04055500
C -34.47242700 3.53210800 0.92520800
C -35.80384000 3.38162100 0.16952800
H -34.40487900 4.57145900 1.24707500
H -34.47804700 2.87841700 1.80584900
C -37.33758100 1.81637000 -0.93062000
H -37.35557400 2.49487800 -1.79158400
H -37.24458900 0.78656800 -1.27072000
C -38.66045000 1.93465000 -0.16082800
C -40.28793000 3.46860700 0.83724600
H -40.23054400 4.49259900 1.20733900
O -36.48027000 4.36839100 -0.14092000
N -36.17359300 2.11018700 -0.10663000
H -35.55281500 1.33678300 0.13138200
O -39.28292200 0.94461700 0.24095800
N -39.10120000 3.19796700 0.03560300
H -38.44628100 3.94983400 -0.17926800
H -40.31363800 2.77423500 1.68503300
C -34.94701600 -3.54942300 -0.64843700
H -35.03267000 -4.60326600 -0.91388600
H -34.95761100 -2.94270100 -1.56199900
C -37.77327100 -1.71275700 1.14136500
H -37.71777300 -2.32399200 2.04970500
H -37.84536700 -0.65972600 1.40607600
C -40.74881000 -3.53984800 -0.41065800
H -40.87597600 -4.61139100 -0.56439100
N -36.08918000 -3.18659300 0.18162900
H -36.74885500 -3.90481600 0.48325200
C -36.48813700 -1.90313100 0.32477200
O -35.85979100 -0.94901600 -0.15277800
N -38.97709000 -2.06000800 0.40092300
H -39.57985800 -1.29685500 0.09200900
O -38.69991400 -4.31060100 0.62484300
C -39.37847600 -3.34320000 0.26034400
C -41.59600000 3.35683600 0.03580100
O -42.22138600 4.36966800 -0.29969200
N -42.00644300 2.10026000 -0.25321200
C -43.12522200 1.83969000 -1.15458300
H -41.42047200 1.30261700 -0.00471200
H -43.06576000 2.52500700 -2.00902700
H -43.02788700 0.81312300 -1.50405200
C -44.51498700 1.97983900 -0.50300300

O -45.27222300 1.01017100 -0.35980900
N -44.83650000 3.23859600 -0.14116200
C -46.08213100 3.58002100 0.52191700
H -44.10432300 3.94177000 -0.25161800
H -45.90719500 3.89205800 1.55893000
H -46.58688700 4.39561000 -0.00720700
H -46.72246400 2.69720800 0.51833300
H -40.75078300 -3.03121600 -1.38267300
N -41.86515700 -3.05105400 0.39187300
H -42.57230100 -3.70643900 0.72631300
C -42.21681700 -1.74606100 0.39949300
O -41.56372300 -0.87103200 -0.18445200
C -43.49539600 -1.42368100 1.18058900
H -43.47859500 -0.36135200 1.42516400
H -43.52884200 -2.01171000 2.10361800
C -45.17178700 -2.94815000 0.26710200
O -44.59556200 -3.92904500 0.75490600
C -46.46909400 -3.09011100 -0.50617700
H -46.31665100 -3.78983000 -1.33364500
H -47.22534900 -3.52696500 0.15390400
H -46.84287400 -2.14023300 -0.89823600
N -44.69269500 -1.68782300 0.40163400
H -45.11673100 -0.87704600 -0.04941400
C -24.23251400 3.37893100 0.18830900
C -25.79390300 1.81751700 -0.88023800
H -25.82126600 2.49058600 -1.74528200
H -25.71250200 0.78522200 -1.21504900
C -27.10354000 1.95195400 -0.09027000
C -28.67749900 3.52859900 0.94307900
H -28.60526400 4.56711900 1.26662500
O -24.90517700 4.36955700 -0.11923600
N -24.61522200 2.10907400 -0.07631500
H -23.99515200 1.33300700 0.15457900
O -27.73433500 0.96781700 0.31736200
N -27.51825400 3.22219700 0.11367600
H -26.85112900 3.95939900 -0.11702500
H -28.67674700 2.87297100 1.82229000
C -26.25452500 -1.78626000 1.04303300
H -26.22455000 -2.45427600 1.91191900
H -26.33802200 -0.75225500 1.37192600
C -29.15627900 -3.51109600 -0.74906700
H -29.23098000 -4.55341900 -1.05960500
N -24.53039800 -3.19309800 0.05182200
H -25.19683100 -3.92997100 0.28600600
C -24.94526000 -1.92243800 0.25276200
O -24.31454200 -0.93934800 -0.15759900
N -27.43363600 -2.08539500 0.24244200
H -28.05549900 -1.31222200 0.00642300
O -27.13873200 -4.34494100 0.29891500
C -27.81450000 -3.35774100 -0.01302300
C -30.01577700 3.38265300 0.19911700
O -30.68809900 4.37283100 -0.11028700
N -30.39724100 2.11250100 -0.06615500
C -31.57290900 1.82091100 -0.87434700
H -29.77632000 1.33709400 0.16468300
H -31.59677700 2.49339600 -1.73995700
H -31.49042600 0.78835500 -1.20818900
C -32.88581700 1.95553900 -0.08985500
O -33.51533300 0.97119200 0.31910400
N -33.30551600 3.22543700 0.10645000
H -32.63905900 3.96351400 -0.12279100
H -29.15971200 -2.86637000 -1.63627400
N -30.31179600 -3.19251900 0.08058000
H -30.97429300 -3.92711600 0.33282100
C -30.72480600 -1.91948700 0.26927300
O -30.09536100 -0.94139300 -0.15506800
C -32.02896800 -1.77342300 1.06593700
H -32.11524600 -0.73329100 1.37403900
H -31.98812800 -2.42311100 1.94821700
C -33.59439000 -3.37451200 0.06299800
O -32.91113900 -4.35217800 0.38857800
N -33.21381500 -2.09541800 0.28285700
H -33.83938400 -1.33005700 0.03105700
H -23.44524500 -4.54041400 -1.09996500
H -23.37457100 -2.84742300 -1.65917200
H -22.89060700 2.86879400 1.80879400
H -22.82004900 4.56301700 1.25326600
C 34.94701600 3.54942300 0.64843700
C 33.59439000 3.37451200 -0.06299800
H 35.03267000 4.60326600 0.91388600
H 34.95761100 2.94270100 1.56199900
C 32.02896800 1.77342300 -1.06593700
H 31.98812800 2.42311100 -1.94821700
H 32.11524600 0.73329100 -1.37403900

C 30.72480600 1.91948700 -0.26927300
C 29.15627900 3.51109600 0.74906700
H 29.23098000 4.55341900 1.05960500
O 32.91113900 4.35217800 -0.38857800
N 33.21381500 2.09541800 -0.28285700
H 33.83938400 1.33005700 -0.03105700
O 30.09536100 0.94139300 0.15506800
N 30.31179600 3.19251900 -0.08058000
H 30.97429300 3.92711600 -0.33282100
H 29.15971200 2.86637000 1.63627400
C 34.47242700 -3.53210800 -0.92520800
H 34.40487900 -4.57145900 -1.24707500
H 34.47804700 -2.87841700 -1.80584900
C 31.57290900 -1.82091100 0.87434700
H 31.59677700 -2.49339600 1.73995700
H 31.49042600 -0.78835500 1.20818900
C 28.67749900 -3.52859900 -0.94307900
H 28.60526400 -4.56711900 -1.26662500
N 33.30551600 -3.22543700 -0.10645000
H 32.63905900 -3.96351400 0.12279100
C 32.88581700 -1.95553900 0.08985500
O 33.51533300 -0.97119200 -0.31910400
N 30.39724100 -2.11250100 0.06615500
H 29.77632000 -1.33709400 -0.16468300
O 30.68809900 -4.37283100 0.11028700
C 30.01577700 -3.38265300 -0.19911700
C 27.81450000 3.35774100 0.01302300
O 27.13873200 4.34494100 -0.29891500
N 27.43363600 2.08539500 -0.24244200
C 26.25452500 1.78626000 -1.04303300
H 28.05549900 1.31222200 -0.00642300
H 26.22455000 2.45427600 -1.91191900
H 26.33802200 0.75225500 -1.37192600
C 24.94526000 1.92243800 -0.25276200
O 24.31454200 0.93934800 0.15759900
N 24.53039800 3.19309800 -0.05182200
C 23.37247200 3.50124600 0.77860800
H 25.19683100 3.92997100 -0.28600600
H 28.67674700 -2.87297100 -1.82229000
N 27.51825400 -3.22219700 -0.11367600
H 26.85112900 -3.95939900 0.11702500
C 27.10354000 -1.95195400 0.09027000
O 27.73433500 -0.96781700 -0.31736200
C 25.79390300 -1.81751700 0.88023800
H 25.71250200 -0.78522200 1.21504900
H 25.82126600 -2.49058600 1.74528200
C 24.23251400 -3.37893100 -0.18830900
O 24.90517700 -4.36955700 0.11923600
C 22.89278900 -3.52454700 -0.92967100
N 24.61522200 -2.10907400 0.07631500
H 23.99515200 -1.33300700 -0.15457900
C 46.46909400 3.09011100 0.50617700
C 45.17178700 2.94815000 -0.26710200
H 47.22534900 3.52696500 -0.15390400
H 46.31665100 3.78983000 1.33364500
H 46.84287400 2.14023300 0.89823600
C 43.49539600 1.42368100 -1.18058900
H 43.52884200 2.01171000 -2.10361800
H 43.47859500 0.36135200 -1.42516400
C 42.21681700 1.74606100 -0.39949300
C 40.74881000 3.53984800 0.41065800
H 40.87597600 4.61139100 0.56439100
O 44.59556200 3.92904500 -0.75490600
N 44.69269500 1.68782300 -0.40163400
H 45.11673100 0.87704600 0.04941400
O 41.56372300 0.87103200 0.18445200
N 41.86515700 3.05105400 -0.39187300
H 42.57230100 3.70643900 -0.72631300
H 40.75078300 3.03121600 1.38267300
C 46.08213100 -3.58002100 -0.52191700
H 46.58688700 -4.39561000 0.00720700
H 45.90719500 -3.89205800 -1.55893000
H 46.72246400 -2.69720800 -0.51833300
C 43.12522200 -1.83969000 1.15458300
H 43.06576000 -2.52500700 2.00902700
H 43.02788700 -0.81312300 1.50405200
C 40.28793000 -3.46860700 -0.83724600
H 40.23054400 -4.49259900 -1.20733900
N 44.83650000 -3.23859600 0.14116200
H 44.10432300 -3.94177000 0.25161800
C 44.51498700 -1.97983900 0.50300300
O 45.27222300 -1.01017100 0.35980900
N 42.00644300 -2.10026000 0.25321200
H 41.42047200 -1.30261700 0.00471200

O 42.22138600 -4.36966800 0.29969200
C 41.59600000 -3.35683600 -0.03580100
C 39.37847600 3.34320000 -0.26034400
O 38.69991400 4.31060100 -0.62484300
N 38.97709000 2.06000800 -0.40092300
C 37.77327100 1.71275700 -1.14136500
H 39.57985800 1.29685500 -0.09200900
H 37.71777300 2.32399200 -2.04970500
H 37.84536700 0.65972600 -1.40607600
C 36.48813700 1.90313100 -0.32477200
O 35.85979100 0.94901600 0.15277800
N 36.08918000 3.18659300 -0.18162900
H 36.74885500 3.90481600 -0.48325200
H 40.31363800 -2.77423500 -1.68503300
N 39.10120000 -3.19796700 -0.03560300
H 38.44628100 -3.94983400 0.17926800
C 38.66045000 -1.93465000 0.16082800
O 39.28292200 -0.94461700 -0.24095800
C 37.33758100 -1.81637000 0.93062000
H 37.24458900 -0.78656800 1.27072000
H 37.35557400 -2.49487800 1.79158400
C 35.80384000 -3.38162100 -0.16952800
O 36.48027000 -4.36839100 0.14092000
N 36.17359300 -2.11018700 0.10663000
H 35.55281500 -1.33678300 -0.13138200
C 11.80662700 3.50592600 0.81332900
C 10.46651000 3.35956500 0.07281900
H 11.87932000 4.54464900 1.13611700
H 11.80927400 2.85084800 1.69296700
C 8.90460900 1.79716800 -0.99356600
H 8.87651000 2.46959300 -1.85908600
H 8.98586300 0.76462700 -1.32765400
C 7.59561400 1.93210600 -0.20263900
C 6.02355300 3.50907800 0.83254200
H 6.09633700 4.54778100 1.15537600
O 9.79339500 4.34984600 -0.23491700
N 10.08381400 2.08947300 -0.19070300
H 10.70403700 1.31362300 0.04055500
O 6.96511200 0.94838000 0.20639700
N 7.18101100 3.20248000 0.00079900
H 7.84818600 3.93948900 -0.23054400
H 6.02635100 2.85394100 1.71213100
C 11.32613800 -3.51851400 -0.89298100
H 11.25338600 -4.55723000 -1.21578000
H 11.32340600 -2.86340900 -1.77259300
C 8.44510700 -1.80656800 0.93314300
H 8.47326500 -2.47901100 1.79864400
H 8.36389300 -0.77403300 1.26726200
C 5.54289000 -3.51520800 -0.87351700
H 5.47008000 -4.55389700 -1.19639300
N 10.16862600 -3.21189200 -0.06131900
H 9.50152100 -3.94891800 0.17014100
C 9.75402100 -1.94151600 0.14209300
O 10.38444600 -0.95779200 -0.26705200
N 7.26581400 -2.09884900 0.13039400
H 6.64577500 -1.32291900 -0.10105200
O 7.55615300 -4.35923600 0.17459100
C 6.88308400 -3.36892900 -0.13313800
C 4.68333300 3.36276300 0.09221500
O 4.01025100 4.35305700 -0.21552800
N 4.30052500 2.09266900 -0.17117000
C 3.12119300 1.80044100 -0.97388300
H 4.92069700 1.31676600 0.06003200
H 3.09300700 2.47292100 -1.83935500
H 3.20236000 0.76791800 -1.30805000
C 1.81234900 1.93541000 -0.18272100
O 1.18197900 0.95170800 0.22657600
N 1.39775500 3.20579800 0.02063200
C 0.24046700 3.51254600 0.85257400
H 2.06486800 3.94278200 -0.21093700
H 5.54010800 -2.86003700 -1.75307500
N 4.38543300 -3.20858100 -0.04179200
H 3.71850200 -3.94564500 0.19003900
C 3.97086800 -1.93820800 0.16168900
O 4.60125500 -0.95450000 -0.24756000
C 2.66207500 -1.80322200 0.95292500
H 2.58081300 -0.77061800 1.28681300
H 2.69045900 -2.47546100 1.81858300
C 1.09991900 -3.36597900 -0.11259000
O 1.77314400 -4.35615500 0.19520100
C -0.24046700 -3.51254600 -0.85257400
N 1.48267100 -2.09579300 0.15043100
H 0.86230100 -1.32004200 -0.08075100
C 22.03264000 3.35408200 0.03778700

C 20.47175500 1.79070000 -1.02865700
H 20.44395500 2.46277500 -1.89446300
H 20.55354600 0.75805400 -1.36226800
C 19.16221700 1.92559600 -0.23861900
C 17.58963200 3.50260600 0.79575500
H 17.66229000 4.54131800 1.11859400
O 21.35928100 4.34399000 -0.27066800
N 21.65041700 2.08372700 -0.22523300
H 22.27081600 1.30822200 0.00677800
O 18.53140200 0.94185600 0.16993700
N 18.74748500 3.19596500 -0.03540800
H 19.41480300 3.93296400 -0.26645700
H 17.59198200 2.84750400 1.67537500
C 20.01113400 -1.81347100 0.89630900
H 20.03901400 -2.48624300 1.76156400
H 19.92976600 -0.78106800 1.23079400
C 17.10950700 -3.52156800 -0.91183900
H 17.03677100 -4.56019800 -1.23491700
N 21.73486900 -3.21833300 -0.09842100
H 21.06779100 -3.95549400 0.13264500
C 21.32033400 -1.94805700 0.10567100
O 21.95098900 -0.96412100 -0.30261000
N 18.83212600 -2.10550500 0.09304300
H 18.21218500 -1.32949800 -0.13840500
O 19.12235000 -4.36592400 0.13674800
C 18.44944800 -3.37549700 -0.17095800
C 16.24971500 3.35632900 0.05487800
O 15.57666500 4.34663600 -0.25292900
N 15.86706900 2.08625800 -0.20884900
C 14.68811800 1.79406200 -1.01213300
H 16.48729800 1.31040100 0.02239600
H 14.66031100 2.46657400 -1.87759600
H 14.76945700 0.76155100 -1.34628900
C 13.37887900 1.92896300 -0.22160700
O 12.74822300 0.94521800 0.18715500
N 12.96424900 3.19933400 -0.01818500
H 13.63157200 3.93631600 -0.24921200
H 17.10704300 -2.86624000 -1.79128200
N 15.95179300 -3.21505200 -0.08042300
H 15.28471800 -3.95212200 0.15096600
C 15.53719200 -1.94469800 0.12310300
O 16.16771200 -0.96093900 -0.28580200
C 14.22815000 -1.80981300 0.91394900
H 14.14686300 -0.77729200 1.24809000
H 14.25619100 -2.48228700 1.77943100
C 12.66627100 -3.37218400 -0.15249900
O 13.33929800 -4.36248900 0.15532900
N 13.04899100 -2.10209900 0.11100500
H 12.42890700 -1.32619900 -0.12042500
H 22.82004900 -4.56301700 -1.25326600
H 22.89060700 -2.86879400 -1.80879400
H 23.37457100 2.84742300 1.65917200
H 23.44524500 4.54041400 1.09996500
N -1.39775500 -3.20579800 -0.02063200
H -2.06486800 -3.94278200 0.21093700
H -0.31329100 -4.55134200 -1.17511100
H -0.24349100 -2.85764800 -1.73233700
H 0.24349100 2.85764800 1.73233700
H 0.31329100 4.55134200 1.17511100

B3LYP_D3

Monomer 8

Zero-point correction= 0.937324 (Hartree/Particle)
Thermal correction to Energy= 1.012092
Thermal correction to Enthalpy= 1.013036
Thermal correction to Gibbs Free Energy= 0.783268
Sum of electronic and zero-point Energies= -3367.889449
Sum of electronic and thermal Energies= -3367.814682
Sum of electronic and thermal Enthalpies= -3367.813737
Sum of electronic and thermal Free Energies= -3368.043506
E(RB3LYP)= -3368.826773

C -0.50845400 -28.79915900 0.00000000
C 0.42396400 -27.59590700 0.00000000
H -0.29143500 -29.41006200 0.88169900

H -1.57100800 -28.53606400 0.00000000
H -0.29143500 -29.41006200 -0.88169900
C 0.58404700 -25.15827800 0.00000000
H 1.24218300 -25.10911500 0.87849400
H 1.24218300 -25.10911500 -0.87849400
C -0.38524300 -23.97326600 0.00000000
C -0.57207600 -21.53728900 0.00000000
H -1.22750100 -21.48972500 -0.87992700
O 1.64554900 -27.71613100 0.00000000
N -0.18263900 -26.37891000 0.00000000
H -1.18871400 -26.26021800 0.00000000
O -1.60654700 -24.12696600 0.00000000
N 0.20087200 -22.75626400 0.00000000
H 1.20826600 -22.63002400 0.00000000
H -1.22750100 -21.48972500 0.87992700
C 0.40160400 -20.35394900 0.00000000
O 1.62123700 -20.51835300 0.00000000
N -0.17955500 -19.13654100 0.00000000
C 0.59562800 -17.91813500 0.00000000
H -1.18669500 -19.00512800 0.00000000
H 1.25031300 -17.87147500 0.88027000
H 1.25031300 -17.87147500 -0.88027000
C -0.38043900 -16.73607200 0.00000000
O -1.59964600 -16.90553000 0.00000000
N 0.19742600 -15.51810600 0.00000000
C -0.57948500 -14.30044200 0.00000000
H 1.20442000 -15.38385800 0.00000000
H -1.23386100 -14.25456300 -0.88043800
H -1.23386100 -14.25456300 0.88043800
C 0.39682600 -13.11820400 0.00000000
C 0.59610200 -10.68243000 0.00000000
H 1.25029700 -10.63633400 0.88050700
H 1.25029700 -10.63633400 -0.88050700
C -0.38143500 -9.50102700 0.00000000
C -0.58296200 -7.06531200 0.00000000
H -1.23709100 -7.01961800 -0.88055200
O 1.61587100 -13.28909700 0.00000000
N -0.18055700 -11.90043400 0.00000000
H -1.18757000 -11.76563700 0.00000000
O -1.60028600 -9.67375900 0.00000000
N 0.19456800 -8.28284700 0.00000000
H 1.20148800 -8.14690100 0.00000000
H -1.23709100 -7.01961800 0.88055200
C 0.39428200 -5.88355800 0.00000000
O 1.61314400 -6.05632200 0.00000000
N -0.18191600 -4.66558700 0.00000000
C 0.59526500 -3.44778000 0.00000000
H -1.18887400 -4.52974500 0.00000000
H 1.24933700 -3.40188700 0.88056900
H 1.24933700 -3.40188700 -0.88056900
C -0.38255700 -2.26645900 0.00000000
O -1.60132800 -2.43995900 0.00000000
N 0.19305200 -1.04827200 0.00000000
H 1.19996400 -0.91196900 0.00000000
C -0.58453800 0.16929800 0.00000000
C 0.39306400 1.35082400 0.00000000
C 0.59486600 3.78653200 0.00000000
H 1.24891900 3.83224000 0.88058400
H 1.24891900 3.83224000 -0.88058400
C -0.38275000 4.96803300 0.00000000
C -0.58452800 7.40374800 0.00000000
H -1.23858700 7.44946600 -0.88058300
O 1.61185800 1.17746400 0.00000000
N -0.18268300 2.56892600 0.00000000
H -1.18960600 2.70515300 0.00000000
O -1.60153200 4.79468900 0.00000000
N 0.19302000 6.18614400 0.00000000
H 1.19994500 6.32230100 0.00000000
H -1.23858700 7.44946600 0.88058300
C 0.39306400 8.58522900 0.00000000
O 1.61184900 8.41197700 0.00000000
N -0.18261500 9.80343500 0.00000000
C 0.59538500 11.02073000 0.00000000
H -1.18950800 9.93969300 0.00000000
H 1.24948900 11.06615200 0.88057400
H 1.24948900 11.06615200 -0.88057400
C -0.38151600 12.20270200 0.00000000
O -1.60037200 12.03037400 0.00000000
N 0.19492300 13.42067700 0.00000000
C -0.58268100 14.63820000 0.00000000
H 1.20187000 13.55619700 0.00000000
H -1.23682000 14.68382100 -0.88055000
H -1.23682000 14.68382100 0.88055000
C 0.39428100 15.81995100 0.00000000

```
C 0.59706800 18.25484400 0.00000000
H 1.25134300 18.29960100 0.88053000
H 1.25134300 18.29960100 -0.88053000
C -0.37785400 19.43803600 0.00000000
C -0.57767300 21.87281100 0.00000000
H -1.23210000 21.91799400 -0.88042400
O 1.61314400 15.64791100 0.00000000
N -0.18186200 17.03826000 0.00000000
H -1.18871800 17.17405100 0.00000000
O -1.59687900 19.26877600 0.00000000
N 0.20048600 20.65576900 0.00000000
H 1.20746000 20.78929400 0.00000000
H -1.23210000 21.91799400 0.88042400
C 0.39662800 23.05574500 0.00000000
O 1.61601100 22.88848400 0.00000000
N -0.18122800 24.27420800 0.00000000
C 0.60132900 25.48744100 0.00000000
H -1.18780700 24.41006400 0.00000000
H 1.25613000 25.52720000 0.88088800
H 1.25613000 25.52720000 -0.88088800
C -0.36616400 26.67830500 0.00000000
O -1.58256200 26.51014700 0.00000000
N 0.23539200 27.89412900 0.00000000
C -0.50534800 29.14672100 0.00000000
H 1.24552200 27.93258900 0.00000000
H -1.56831200 28.90315700 0.00000000
H -0.27262000 29.73829800 0.89233900
H -0.27262000 29.73829800 -0.89233900
H -1.23859500 0.21500300 -0.88058300
H -1.23859500 0.21500300 0.88058300
```

B3LYP_D3

Dimer 8

Zero-point correction= 1.911698 (Hartree/Particle)

Thermal correction to Energy= 2.047200

Thermal correction to Enthalpy= 2.048144

Thermal correction to Gibbs Free Energy= 1.708613

Sum of electronic and zero-point Energies= -6736.004644

Sum of electronic and thermal Energies= -6735.869141

Sum of electronic and thermal Enthalpies= -6735.868197

Sum of electronic and thermal Free Energies= -6736.207729

Electronic Energy (EE)= -6737.916342

```
C 11.81178700 3.55458700 0.79894400
C 10.46005000 3.39161700 0.08299600
H 11.89667400 4.60363700 1.08297400
H 11.82163000 2.93189800 1.70170400
C 8.89632100 1.80763800 -0.94932600
H 8.85610900 2.47260700 -1.82017700
H 8.98335400 0.77307600 -1.27543800
C 7.59130300 1.93922900 -0.15154600
C 6.02087100 3.51208500 0.89261600
H 6.09474900 4.54882100 1.22151300
O 9.77682900 4.37456500 -0.22632900
N 10.08022000 2.11640900 -0.15952400
H 10.70577000 1.34699700 0.07964200
O 6.96187900 0.95361300 0.25502900
N 7.17753400 3.20858800 0.05892900
H 7.83985700 3.94780100 -0.17987100
H 6.02351000 2.85186300 1.76835100
C 11.34191500 -3.49814800 -0.89793600
H 11.27517200 -4.53178400 -1.23786900
H 11.34812400 -2.82926300 -1.76708700
C 8.43964400 -1.81967500 0.92778100
H 8.46288300 -2.50690400 1.78175400
H 8.35618800 -0.79304200 1.27918000
C 5.54735400 -3.49748900 -0.92212300
H 5.47608200 -4.53034800 -1.26351800
N 10.17404200 -3.20625200 -0.07516200
H 9.50755500 -3.94843900 0.14036200
C 9.75355200 -1.94012200 0.14265700
O 10.38311500 -0.94857700 -0.24846500
N 7.26508800 -2.09799300 0.11332100
H 6.64393800 -1.31909800 -0.10483800
O 7.55726800 -4.35857500 0.11887100
C 6.88467100 -3.36361200 -0.17419100
C 4.68007600 3.37113900 0.15230200
```

O 4.00431500 4.36341800 -0.14309000
N 4.30008800 2.10332600 -0.12596400
C 3.12200800 1.81777700 -0.93301200
H 4.92188300 1.32634200 0.09734100
H 3.09264500 2.50104100 -1.78997500
H 3.20640700 0.78979400 -1.28005300
C 1.81180700 1.93926800 -0.14190400
O 1.18111700 0.94872200 0.25016500
N 1.39616100 3.20594800 0.08122500
C 0.23699000 3.49874400 0.91544800
H 2.06230100 3.94724900 -0.13934800
H 5.54734300 -2.82689200 -1.78997600
N 4.38692000 -3.20594400 -0.08905400
H 3.71985600 -3.94733800 0.12810200
C 3.97116600 -1.93960700 0.13599200
O 4.60203800 -0.94829400 -0.25377500
C 2.66029500 -1.81946900 0.92622900
H 2.57748300 -0.79297700 1.27808200
H 2.68708800 -2.50687100 1.77994700
C 1.10170200 -3.36427600 -0.17018500
O 1.77497600 -4.35926800 0.12162200
C -0.23699000 -3.49874400 -0.91544800
N 1.48295100 -2.09864000 0.11596100
H 0.86248500 -1.31941600 -0.10301500
C 23.33408800 3.10534200 0.66173000
C 22.03773700 2.97596900 -0.11536000
H 24.09058100 3.55471800 0.01039000
H 23.18006700 3.78996100 1.50144900
H 23.70844200 2.14897500 1.03711400
C 20.36366700 1.46636800 -1.05751400
H 20.39763700 2.07064400 -1.96997300
H 20.34822000 0.40851000 -1.32078600
C 19.08382900 1.77373100 -0.27242300
C 17.61359100 3.55166800 0.56805400
H 17.73982400 4.62035300 0.74122800
O 21.46121300 3.96487400 -0.58635000
N 21.55983300 1.71783100 -0.27261800
H 21.98420600 0.89957600 0.16432300
O 18.43056300 0.88788400 0.29477200
N 18.73128400 3.07814800 -0.24171500
H 19.43830700 3.73993800 -0.56356800
H 17.61469400 3.02560600 1.53074600
C 22.95080400 -3.54768500 -0.48682400
H 23.45620100 -4.37181600 0.02826900
H 22.77621400 -3.84201900 -1.52905800
H 23.59039900 -2.66452900 -0.46802900
C 19.99207900 -1.83867800 1.21839600
H 19.93249700 -2.53902300 2.06054900
H 19.89399000 -0.81848400 1.58585400
C 17.15695800 -3.43364800 -0.80374200
H 17.10024100 -4.45105300 -1.19167300
N 21.70483800 -3.21871200 0.18189200
H 20.97300600 -3.92412000 0.27965500
C 21.38221200 -1.96655800 0.56508700
O 22.13869700 -0.99399000 0.43869900
N 18.87397300 -2.08387800 0.31190000
H 18.28780100 -1.28227500 0.07699700
O 19.09004800 -4.35364600 0.31858100
C 18.46438400 -3.33537700 0.00051100
C 16.24424200 3.36623300 -0.10812900
O 15.56567000 4.33958600 -0.45639500
N 15.84366400 2.08552900 -0.27177500
C 14.64086700 1.75082100 -1.01961800
H 16.44646100 1.31733700 0.02432000
H 14.58615200 2.37787300 -1.91716200
H 14.71373200 0.70262300 -1.30266400
C 13.35472900 1.92630000 -0.20127100
O 12.72629400 0.96368700 0.25877300
N 12.95505300 3.20688200 -0.03606500
H 13.61471000 3.93057800 -0.32432000
H 17.18307800 -2.72460000 -1.63928500
N 15.96949800 -3.17745900 0.00155900
H 15.31455800 -3.93315300 0.20253500
C 15.52818600 -1.91791500 0.21974500
O 16.15076000 -0.92079400 -0.16397100
C 14.20450300 -1.81356800 0.99015300
H 14.11069700 -0.78984500 1.34792200
H 14.22197800 -2.50686000 1.83927100
C 12.67251800 -3.36021300 -0.13844100
O 13.34909300 -4.35193300 0.15550800
N 13.04146100 -2.09359900 0.16004600
H 12.42050500 -1.31647800 -0.06506200
C -11.34191500 3.49814800 0.89793600
C -12.67251800 3.36021300 0.13844100

H -11.27517200 4.53178400 1.23786900
H -11.34812400 2.82926300 1.76708700
C -14.20450300 1.81356800 -0.99015300
H -14.22197800 2.50686000 -1.83927100
H -14.11069700 0.78984500 -1.34792200
C -15.52818600 1.91791500 -0.21974500
C -17.15695800 3.43364800 0.80374200
H -17.10024100 4.45105300 1.19167300
O -13.34909300 4.35193300 -0.15550800
N -13.04146100 2.09359900 -0.16004600
H -12.42050500 1.31647800 0.06506200
O -16.15076000 0.92079400 0.16397100
N -15.96949800 3.17745900 -0.00155900
H -15.31455800 3.93315300 -0.20253500
H -17.18307800 2.72460000 1.63928500
C -11.81178700 -3.55458700 -0.79894400
H -11.89667400 -4.60363700 -1.08297400
H -11.82163000 -2.93189800 -1.70170400
C -14.64086700 -1.75082100 1.01961800
H -14.58615200 -2.37787300 1.91716200
H -14.71373200 -0.70262300 1.30266400
C -17.61359100 -3.55166800 -0.56805400
H -17.73982400 -4.62035300 -0.74122800
N -12.95505300 -3.20688200 0.03606500
H -13.61471000 -3.93057800 0.32432000
C -13.35472900 -1.92630000 0.20127100
O -12.72629400 -0.96368700 -0.25877300
N -15.84366400 -2.08552900 0.27177500
H -16.44646100 -1.31733700 -0.02432000
O -15.56567000 -4.33958600 0.45639500
C -16.24424200 -3.36623300 0.10812900
C -18.46438400 3.33537700 -0.00051100
O -19.09004800 4.35364600 -0.31858100
N -18.87397300 2.08387800 -0.31190000
C -19.99207900 1.83867800 -1.21839600
H -18.28780100 1.28227500 -0.07699700
H -19.93249700 2.53902300 -2.06054900
H -19.89399000 0.81848400 -1.58585400
C -21.38221200 1.96655800 -0.56508700
O -22.13869700 0.99399000 -0.43869900
N -21.70483800 3.21871200 -0.18189200
C -22.95080400 3.54768500 0.48682400
H -20.97300600 3.92412000 -0.27965500
H -22.77621400 3.84201900 1.52905800
H -23.45620100 4.37181600 -0.02826900
H -23.59039900 2.66452900 0.46802900
H -17.61469400 -3.02560600 -1.53074600
N -18.73128400 -3.07814800 0.24171500
H -19.43830700 -3.73993800 0.56356800
C -19.08382900 -1.77373100 0.27242300
O -18.43056300 -0.88788400 -0.29477200
C -20.36366700 -1.46636800 1.05751400
H -20.34822000 -0.40851000 1.32078600
H -20.39763700 -2.07064400 1.96997300
C -22.03773700 -2.97596900 0.11536000
O -21.46121300 -3.96487400 0.58635000
C -23.33408800 -3.10534200 -0.66173000
H -23.18006700 -3.78996100 -1.50144900
H -24.09058100 -3.55471800 -0.01039000
H -23.70844200 -2.14897500 -1.03711400
N -21.55983300 -1.71783100 0.27261800
H -21.98420600 -0.89957600 -0.16432300
C -1.10170200 3.36427600 0.17018500
C -2.66029500 1.81946900 -0.92622900
H -2.68708800 2.50687100 -1.77994700
H -2.57748300 0.79297700 -1.27808200
C -3.97116600 1.93960700 -0.13599200
C -5.54735400 3.49748900 0.92212300
H -5.47608200 4.53034800 1.26351800
O -1.77497600 4.35926800 -0.12162200
N -1.48295100 2.09864000 -0.11596100
H -0.86248500 1.31941600 0.10301500
O -4.60203800 0.94829400 0.25377500
N -4.38692000 3.20594400 0.08905400
H -3.71985600 3.94733800 -0.12810200
H -5.54734300 2.82689200 1.78997600
C -3.12200800 -1.81777700 0.93301200
H -3.09264500 -2.50104100 1.78997500
H -3.20640700 -0.78979400 1.28005300
C -6.02087100 -3.51208500 -0.89261600
H -6.09474900 -4.54882100 -1.22151300
N -1.39616100 -3.20594800 -0.08122500
H -2.06230100 -3.94724900 0.13934800
C -1.81180700 -1.93926800 0.14190400

O -1.18111700 -0.94872200 -0.25016500
N -4.30008800 -2.10332600 0.12596400
H -4.92188300 -1.32634200 -0.09734100
O -4.00431500 -4.36341800 0.14309000
C -4.68007600 -3.37113900 -0.15230200
C -6.88467100 3.36361200 0.17419100
O -7.55726800 4.35857500 -0.11887100
N -7.26508800 2.09799300 -0.11332100
C -8.43964400 1.81967500 -0.92778100
H -6.64393800 1.31909800 0.10483800
H -8.46288300 2.50690400 -1.78175400
H -8.35618800 0.79304200 -1.27918000
C -9.75355200 1.94012200 -0.14265700
O -10.38311500 0.94857700 0.24846500
N -10.17404200 3.20625200 0.07516200
H -9.50755500 3.94843900 -0.14036200
H -6.02351000 -2.85186300 -1.76835100
N -7.17753400 -3.20858800 -0.05892900
H -7.83985700 -3.94780100 0.17987100
C -7.59130300 -1.93922900 0.15154600
O -6.96187900 -0.95361300 -0.25502900
C -8.89632100 -1.80763800 0.94932600
H -8.98335400 -0.77307600 1.27543800
H -8.85610900 -2.47260700 1.82017700
C -10.46005000 -3.39161700 -0.08299600
O -9.77682900 -4.37456500 0.22632900
N -10.08022000 -2.11640900 0.15952400
H -10.70577000 -1.34699700 -0.07964200
H -0.30887600 -4.53201800 -1.25547700
H -0.23818200 -2.82925500 -1.78415300
H 0.23818200 2.82925500 1.78415300
H 0.30887600 4.53201800 1.25547700

B3LYP_D3

Monomer 12

Zero-point correction= 1.382512 (Hartree/Particle)
Thermal correction to Energy= 1.493666
Thermal correction to Enthalpy= 1.494611
Thermal correction to Gibbs Free Energy= 1.164070
Sum of electronic and zero-point Energies= -5031.603926
Sum of electronic and thermal Energies= -5031.492771
Sum of electronic and thermal Enthalpies= -5031.491827
Sum of electronic and thermal Free Energies= -5031.822368
E(RB3LYP)= -5032.986438

C -0.58006900 -14.29655200 0.00000000
C 0.40025400 -13.11723500 0.00000000
C 0.60651600 -10.68149900 0.00000000
H 1.26062200 -10.63673100 0.88058500
H 1.26062200 -10.63673100 -0.88058500
C -0.36969500 -9.49867900 0.00000000
C -0.56819700 -7.06244400 0.00000000
H -1.22216100 -7.01569800 -0.88058300
O 1.61864100 -13.29351200 0.00000000
N -0.17291200 -11.89796100 0.00000000
H -1.17959200 -11.75958600 0.00000000
O -1.58870200 -9.67080400 0.00000000
N 0.20747100 -8.28132700 0.00000000
H 1.21459200 -8.14614700 0.00000000
H -1.22216100 -7.01569800 0.88058300
C 0.41157200 -5.88259300 0.00000000
O 1.63004200 -6.05839500 0.00000000
N -0.16210000 -4.66360900 0.00000000
C 0.61677000 -3.44677300 0.00000000
H -1.16884600 -4.52563300 0.00000000
H 1.27082900 -3.40167700 0.88059700
H 1.27082900 -3.40167700 -0.88059700
C -0.36013100 -2.26453000 0.00000000
O -1.57902000 -2.43748100 0.00000000
N 0.21633300 -1.04687500 0.00000000
C -0.55992900 0.17164500 0.00000000
H 1.22338200 -0.91111900 0.00000000
H -1.21390700 0.21806800 -0.88058500
H -1.21390700 0.21806800 0.88058500
C 0.41931200 1.35197700 0.00000000
C 0.62341400 3.78789600 0.00000000
H 1.27744500 3.83329800 0.88060100

H 1.27744500 3.83329800 -0.88060100
C -0.35406000 4.96966100 0.00000000
C -0.55521900 7.40567000 0.00000000
H -1.20922200 7.45171700 -0.88058800
O 1.63786900 1.17671400 0.00000000
N -0.15489900 2.57069500 0.00000000
H -1.16171300 2.70823000 0.00000000
O -1.57285700 4.79610200 0.00000000
N 0.22176300 6.18761400 0.00000000
H 1.22873300 6.32390600 0.00000000
H -1.20922200 7.45171700 0.88058800
C 0.42333700 8.58653400 0.00000000
O 1.64198900 8.41197500 0.00000000
N -0.15163000 9.80491100 0.00000000
C 0.62591000 11.02260400 0.00000000
H -1.15853100 9.94180100 0.00000000
H 1.27992000 11.06840800 0.88059600
H 1.27992000 11.06840800 -0.88059600
C -0.35225900 12.20380400 0.00000000
O -1.57096100 12.02961200 0.00000000
N 0.22278400 13.42215400 0.00000000
H 1.22965400 13.55910500 0.00000000
C -0.55519200 14.63955900 0.00000000
C 0.42232800 15.82119400 0.00000000
C 0.62290600 18.25736000 0.00000000
H 1.27689900 18.30365600 0.88059100
H 1.27689900 18.30365600 -0.88059100
C -0.35604100 19.43785200 0.00000000
C -0.56088800 21.87333400 0.00000000
H -1.21501900 21.91819300 -0.88056600
O 1.64111400 15.64776200 0.00000000
N -0.15371000 17.03910500 0.00000000
H -1.16071500 17.17507600 0.00000000
O -1.57462200 19.26298200 0.00000000
N 0.21819500 20.65666700 0.00000000
H 1.22495200 20.79427400 0.00000000
H -1.21501900 21.91819300 0.88056600
C 0.41525000 23.05602600 0.00000000
O 1.63423800 22.88429800 0.00000000
N -0.16210000 24.27343000 0.00000000
C 0.61380500 25.49207700 0.00000000
H -1.16920600 24.40829100 0.00000000
H 1.26782000 25.53871900 0.88057500
H 1.26782000 25.53871900 -0.88057500
C -0.36547000 26.67209500 0.00000000
O -1.58396300 26.49722200 0.00000000
N 0.20847300 27.89125700 0.00000000
C -0.57152900 29.10727200 0.00000000
H 1.21514800 28.02896700 0.00000000
H -1.22578100 29.15158900 -0.88053100
H -1.22578100 29.15158900 0.88053100
C 0.40312900 30.29093600 0.00000000
C 0.60100800 32.72631600 0.00000000
H 1.25522700 32.77236300 0.88050500
H 1.25522700 32.77236300 -0.88050500
C -0.37626200 33.90762400 0.00000000
C -0.58007300 36.34232500 0.00000000
H -1.23457800 36.38661000 -0.88041600
O 1.62233100 30.12130200 0.00000000
N -0.17542100 31.50812400 0.00000000
H -1.18254700 31.64203700 0.00000000
O -1.59496700 33.73613100 0.00000000
N 0.19987300 35.12641400 0.00000000
H 1.20663100 35.26175200 0.00000000
H -1.23457800 36.38661000 0.88041600
C 0.39272000 37.52654400 0.00000000
O 1.61232000 37.36084300 0.00000000
N -0.18646000 38.74438300 0.00000000
C 0.59492000 39.95839300 0.00000000
H -1.19318300 38.87931000 0.00000000
H 1.24969200 39.99877900 0.88087800
H 1.24969200 39.99877900 -0.88087800
C -0.37360700 41.14838000 0.00000000
O -1.58986100 40.97918900 0.00000000
N 0.22703400 42.36469200 0.00000000
C -0.51457700 43.61673700 0.00000000
H 1.23714400 42.40383600 0.00000000
H -0.28218200 44.20848300 0.89231400
H -0.28218200 44.20848300 -0.89231400
H -1.57739300 43.37253900 0.00000000
H -1.20924200 14.68507200 -0.88058500
H -1.20924200 14.68507200 0.88058500
C -0.58527300 -43.26530300 0.00000000
C 0.34969100 -42.06406900 0.00000000

C 0.51484800 -39.62675100 0.00000000
H 1.17311600 -39.57904600 0.87847100
H 1.17311600 -39.57904600 -0.87847100
C -0.45182500 -38.43961300 0.00000000
C -0.63225100 -36.00287100 0.00000000
H -1.28754500 -35.95337700 -0.87992200
O 1.57102700 -42.18687500 0.00000000
N -0.25436300 -40.84582100 0.00000000
H -1.26018900 -40.72495200 0.00000000
O -1.67347500 -38.59048100 0.00000000
N 0.13725000 -37.22402800 0.00000000
H 1.14498000 -37.10028800 0.00000000
H -1.28754500 -35.95337700 0.87992200
C 0.34504500 -34.82248900 0.00000000
O 1.56416500 -34.99065200 0.00000000
N -0.23225200 -33.60323200 0.00000000
C 0.54683800 -32.38729700 0.00000000
H -1.23896200 -33.46847200 0.00000000
H 1.20171600 -32.34280800 0.88024200
H 1.20171600 -32.34280800 -0.88024200
C -0.42532700 -31.20199700 0.00000000
O -1.64510200 -31.36731300 0.00000000
N 0.15665900 -29.98596500 0.00000000
C -0.61591500 -28.76552900 0.00000000
H 1.16412200 -29.85508100 0.00000000
H -1.27015200 -28.71733000 -0.88041900
H -1.27015200 -28.71733000 0.88041900
C 0.36460100 -27.58673400 0.00000000
C 0.57206000 -25.15146100 0.00000000
H 1.22640700 -25.10742500 0.88050000
H 1.22640700 -25.10742500 -0.88050000
C -0.40189200 -23.96711600 0.00000000
C -0.59616600 -21.53075700 0.00000000
H -1.25018400 -21.48313700 -0.88053200
O 1.58303700 -27.76188300 0.00000000
N -0.20855200 -26.36695200 0.00000000
H -1.21511000 -26.22863500 0.00000000
O -1.62124500 -24.13625500 0.00000000
N 0.17771200 -22.75063600 0.00000000
H 1.18502900 -22.61753500 0.00000000
H -1.25018400 -21.48313700 0.88053200
C 0.38461400 -20.35190600 0.00000000
O 1.60295800 -20.52828400 0.00000000
N -0.18815300 -19.13231300 0.00000000
C 0.59195900 -17.91634500 0.00000000
H -1.19476100 -18.99363600 0.00000000
H 1.24614100 -17.87197000 0.88056300
H 1.24614100 -17.87197000 -0.88056300
C -0.38329100 -16.73282200 0.00000000
O -1.60244100 -16.90377700 0.00000000
N 0.19485600 -15.51586200 0.00000000
H 1.20206000 -15.38149900 0.00000000
H -1.64726000 -42.99994700 0.00000000
H -0.36959000 -43.87669200 0.88168900
H -1.23403400 -14.24942000 0.88057200
H -1.23403400 -14.24942000 -0.88057200
H -0.36959000 -43.87669200 -0.88168900

B3LYP_D3

Dimer 12

Zero-point correction= 2.823921 (Hartree/Particle)
Thermal correction to Energy= 3.022331
Thermal correction to Enthalpy= 3.023275
Thermal correction to Gibbs Free Energy= 2.539039
Sum of electronic and zero-point Energies= -10063.541379
Sum of electronic and thermal Energies= -10063.342969
Sum of electronic and thermal Enthalpies= -10063.342025
Sum of electronic and thermal Free Energies= -10063.826261
Electronic Energy (EE)= -10066.365300

C -22.85436600 3.18800900 -1.63480200
C -24.18681600 2.57283100 -2.09114900
H -22.78507400 4.17654600 -2.08895600
H -22.85089400 3.28486000 -0.54239800
C -25.72189300 0.68012700 -1.86119300
H -25.73891500 0.60482100 -2.95485000
H -25.63769000 -0.31121700 -1.42025100
C -27.03373900 1.30276000 -1.36978100
C -28.65689600 3.10573400 -1.66681000

H -28.59801100 4.11790400 -2.06762300
O -24.86092600 3.09500500 -2.98603600
N -24.55579500 1.44420600 -1.44430000
H -23.93942900 1.02107700 -0.75079900
O -27.65315000 0.85894000 -0.39546500
N -27.46929000 2.36840000 -2.07643400
H -26.81895800 2.77137300 -2.75025600
H -28.69437500 3.14387200 -0.57202200
C -23.22411800 -3.07833100 1.80245800
H -23.30680600 -4.04014200 2.30866900
H -23.23473300 -3.23303900 0.71688500
C -26.04215700 -0.51318800 1.89284400
H -25.98845200 -0.35504400 2.97613600
H -26.11271900 0.44361800 1.37981500
C -29.01014100 -2.90995600 1.98600900
H -29.16061900 -3.77482400 2.63219100
N -24.36374900 -2.25204800 2.17843100
H -25.01412700 -2.57168600 2.89656600
C -24.76589000 -1.21339600 1.41489200
O -24.14984000 -0.85001900 0.40390600
N -27.24249000 -1.27120500 1.57642900
H -27.82902200 -0.92532300 0.81729400
O -26.96159600 -2.77084400 3.26847000
C -27.63984300 -2.31204200 2.34184600
C -29.95034400 2.48029200 -2.21162200
O -30.56972400 3.01647400 -3.13782900
N -30.35039500 1.33322800 -1.61424400
C -31.43491700 0.51963300 -2.15766600
H -29.75585100 0.88494100 -0.91589100
H -31.34168000 0.47574900 -3.25011000
H -31.32705900 -0.48030200 -1.74075200
C -32.84608200 1.02485600 -1.80197900
O -33.61797000 0.36216000 -1.09461700
N -33.16975500 2.21444600 -2.34569600
H -32.43272500 2.69047400 -2.86776600
H -29.00200400 -3.23319000 0.93791400
N -30.10795500 -1.97238900 2.18853200
H -30.79963900 -2.14388500 2.91957600
C -30.44586000 -1.06740700 1.24520700
O -29.80809200 -0.92589500 0.19257400
C -31.68666600 -0.22885700 1.56506200
H -31.66511000 0.65781400 0.93016500
H -31.67421000 0.07353800 2.61811100
C -33.38466100 -1.88660500 2.14931100
O -32.78020600 -2.19936200 3.18395400
N -32.91203700 -0.95619800 1.28642300
H -33.37454000 -0.71920000 0.40777000
C -12.67008600 2.54036900 -2.12831100
C -14.22478600 0.66816500 -1.85307100
H -14.25335400 0.57557600 -2.94512500
H -14.14704800 -0.31652100 -1.39671100
C -15.52348300 1.31641800 -1.35863000
C -17.09076300 3.17550000 -1.66095000
H -17.02021900 4.16001300 -2.12353400
O -13.34178900 3.05189200 -3.03151000
N -13.04671400 1.42706900 -1.46022200
H -12.43292500 1.01296800 -0.75927500
O -16.14237500 0.89159800 -0.37426400
N -15.94017600 2.38278700 -2.07497200
H -15.28195800 2.75883900 -2.75747600
H -17.08149100 3.28139400 -0.56942400
C -14.57229500 -0.61379800 1.89431900
H -14.54611400 -0.52134400 2.98646200
H -14.65093300 0.37081000 1.43797900
C -17.45954800 -3.10530800 1.72100200
H -17.53070600 -4.08758700 2.18823700
C -13.27160800 -1.25991600 1.40246900
O -12.65191000 -0.83498900 0.41866800
N -15.74818300 -1.37474500 1.49893500
H -16.36211900 -0.96124100 0.79772500
O -15.45254800 -3.00001200 3.06972300
C -16.12390300 -2.48907400 2.16591200
C -18.42829300 2.56248500 -2.10496100
O -19.10245600 3.07896700 -3.00345000
N -18.80306800 1.44541900 -1.44213000
C -19.98139500 0.68825600 -1.83734200
H -18.18710200 1.02735900 -0.74544500
H -20.01117200 0.60109200 -2.92982500
H -19.90278800 -0.29872200 -1.38608100
C -21.28018400 1.33293100 -1.33853200
O -21.89769200 0.90189100 -0.35605000
N -21.69916300 2.40214800 -2.04947400
H -21.04209800 2.78275900 -2.73041800
H -17.46632500 -3.21652100 0.63002300

N -18.61098400 -2.31092900 2.12858200
H -19.26582600 -2.67874500 2.81903300
C -19.02821800 -1.24927200 1.40560200
O -18.41163800 -0.83172800 0.41645100
C -20.32456300 -0.59653000 1.89962000
H -20.40730100 0.38170100 1.43062200
H -20.28793600 -0.48905100 2.99007300
C -21.87914700 -2.46203400 2.21890100
O -21.20001700 -2.95978000 3.12433500
N -21.50493500 -1.36218800 1.52715800
H -22.12099400 -0.96371900 0.81902700
C 23.22411800 3.07833100 -1.80245800
C 21.87914700 2.46203400 -2.21890100
H 23.30680600 4.04014200 -2.30866900
H 23.23473300 3.23303900 -0.71688500
C 20.32456300 0.59653000 -1.89962000
H 20.28793600 0.48905100 -2.99007300
H 20.40730100 -0.38170100 -1.43062200
C 19.02821800 1.24927200 -1.40560200
C 17.45954800 3.10530800 -1.72100200
H 17.53070600 4.08758700 -2.18823700
O 21.20001700 2.95978000 -3.12433500
N 21.50493500 1.36218800 -1.52715800
H 22.12099400 0.96371900 -0.81902700
O 18.41163800 0.83172800 -0.41645100
N 18.61098400 2.31092900 -2.12858200
H 19.26582600 2.67874500 -2.81903300
H 17.46632500 3.21652100 -0.63002300
C 22.85436600 -3.18800900 1.63480200
H 22.78507400 -4.17654600 2.08895600
H 22.85089400 -3.28486000 0.54239800
C 19.98139500 -0.68825600 1.83734200
H 20.01117200 -0.60109200 2.92982500
H 19.90278800 0.29872200 1.38608100
C 17.09076300 -3.17550000 1.66095000
H 17.02021900 -4.16001300 2.12353400
N 21.69916300 -2.40214800 2.04947400
H 21.04209800 -2.78275900 2.73041800
C 21.28018400 -1.33293100 1.33853200
O 21.89769200 -0.90189100 0.35605000
N 18.80306800 -1.44541900 1.44213000
H 18.18710200 -1.02735900 0.74544500
O 19.10245600 -3.07896700 3.00345000
C 18.42829300 -2.56248500 2.10496100
C 16.12390300 2.48907400 -2.16591200
O 15.45254800 3.00001200 -3.06972300
N 15.74818300 1.37474500 -1.49893500
C 14.57229500 0.61379800 -1.89431900
H 16.36211900 0.96124100 -0.79772500
H 14.54611400 0.52134400 -2.98646200
H 14.65093300 -0.37081000 -1.43797900
C 13.27160800 1.25991600 -1.40246900
O 12.65191000 0.83498900 -0.41866800
N 12.85423500 2.32488000 -2.12048600
C 11.70118500 3.11540500 -1.70952800
H 13.51343800 2.70201700 -2.80151400
H 17.08149100 -3.28139400 0.56942400
N 15.94017600 -2.38278700 2.07497200
H 15.28195800 -2.75883900 2.75747600
C 15.52348300 -1.31641800 1.35863000
O 16.14237500 -0.89159800 0.37426400
C 14.22478600 -0.66816500 1.85307100
H 14.14704800 0.31652100 1.39671100
H 14.25335400 -0.57557600 2.94512500
C 12.67008600 -2.54036900 2.12831100
O 13.34178900 -3.05189200 3.03151000
C 11.33374600 -3.15596500 1.68429100
N 13.04671400 -1.42706900 1.46022200
H 12.43292500 -1.01296800 0.75927500
C 33.38466100 1.88660500 -2.14931100
C 31.68666600 0.22885700 -1.56506200
H 31.67421000 -0.07353800 -2.61811100
H 31.66511000 -0.65781400 -0.93016500
C 30.44586000 1.06740700 -1.24520700
C 29.01014100 2.90995600 -1.98600900
H 29.16061900 3.77482400 -2.63219100
O 32.78020600 2.19936200 -3.18395400
N 32.91203700 0.95619800 -1.28642300
H 33.37454000 0.71920000 -0.40777000
O 29.80809200 0.92589500 -0.19257400
N 30.10795500 1.97238900 -2.18853200
H 30.79963900 2.14388500 -2.91957600
H 29.00200400 3.23319000 -0.93791400
C 31.43491700 -0.51963300 2.15766600

H 31.34168000 -0.47574900 3.25011000
H 31.32705900 0.48030200 1.74075200
C 28.65689600 -3.10573400 1.66681000
H 28.59801100 -4.11790400 2.06762300
N 33.16975500 -2.21444600 2.34569600
H 32.43272500 -2.69047400 2.86776600
C 32.84608200 -1.02485600 1.80197900
O 33.61797000 -0.36216000 1.09461700
N 30.35039500 -1.33322800 1.61424400
H 29.75585100 -0.88494100 0.91589100
O 30.56972400 -3.01647400 3.13782900
C 29.95034400 -2.48029200 2.21162200
C 27.63984300 2.31204200 -2.34184600
O 26.96159600 2.77084400 -3.26847000
N 27.24249000 1.27120500 -1.57642900
C 26.04215700 0.51318800 -1.89284400
H 27.82902200 0.92532300 -0.81729400
H 25.98845200 0.35504400 -2.97613600
H 26.11271900 -0.44361800 -1.37981500
C 24.76589000 1.21339600 -1.41489200
O 24.14984000 0.85001900 -0.40390600
N 24.36374900 2.25204800 -2.17843100
H 25.01412700 2.57168600 -2.89656600
H 28.69437500 -3.14387200 0.57202200
N 27.46929000 -2.36840000 2.07643400
H 26.81895800 -2.77137300 2.75025600
C 27.03373900 -1.30276000 1.36978100
O 27.65315000 -0.85894000 0.39546500
C 25.72189300 -0.68012700 1.86119300
H 25.63769000 0.31121700 1.42025100
H 25.73891500 -0.60482100 2.95485000
C 24.18681600 -2.57283100 2.09114900
O 24.86092600 -3.09500500 2.98603600
N 24.55579500 -1.44420600 1.44430000
H 23.93942900 -1.02107700 0.75079900
C 0.18474200 3.12950200 -1.70022900
C -1.15018600 2.51111000 -2.14474800
H 0.25376300 4.11206800 -2.16717000
H 0.19147000 3.24039600 -0.60917300
C -2.70281400 0.63749200 -1.86690700
H -2.72857000 0.54064600 -2.95868500
H -2.62478800 -0.34536000 -1.40658600
C -4.00360800 1.28577400 -1.37811200
C -5.57464500 3.13948500 -1.69398000
H -5.50443400 4.12189500 -2.16105900
O -1.82078500 3.01900600 -3.05084500
N -1.52680500 1.39948800 -1.47383900
H -0.91316500 0.98761900 -0.77142900
O -4.62252100 0.86580700 -0.39153300
N -4.42163900 2.34692300 -2.10155800
H -3.76228800 2.72130400 -2.78398700
H -5.56844900 3.25062600 -0.60295000
C -0.18474200 -3.12950200 1.70022900
H -0.25376300 -4.11206800 2.16717000
H -0.19147000 -3.24039600 0.60917300
C -3.05786600 -0.62929600 1.87515200
H -3.03230100 -0.53336000 2.96701300
H -3.13655200 0.35387000 1.41561100
C -5.94307600 -3.12349300 1.70630300
H -6.01144700 -4.10697100 2.17139800
N -1.33816500 -2.33795100 2.10857900
H -1.99736500 -2.71333900 2.79060200
C -1.75663400 -1.27635000 1.38604900
O -1.13769500 -0.85505000 0.40008500
N -4.23316000 -1.39196300 1.48134800
H -4.84747000 -0.97971400 0.77974100
O -3.93673000 -3.01351600 3.05583200
C -4.60837500 -2.50486300 2.15096000
C -6.90999300 2.52255500 -2.13928600
O -7.57956800 3.03157100 -3.04549700
N -7.28807000 1.41096200 -1.46920400
C -8.46512300 0.65079000 -1.86270000
H -6.67522900 0.99806000 -0.76672300
H -8.49134100 0.55521700 -2.95454700
H -8.38796900 -0.33263900 -1.40356700
C -9.76509100 1.29982500 -1.37261100
O -10.38496700 0.87853400 -0.38733600
N -10.18190000 2.36282800 -2.09383100
C -11.33374600 3.15596500 -1.68429100
H -9.52251900 2.73743900 -2.77604300
H -5.95032700 -3.23222500 0.61503500
N -7.09643000 -2.33297500 2.11661000
H -7.75609000 -2.71017200 2.79712300
C -7.51389100 -1.26903000 1.39716700

O -6.89341900 -0.84492200 0.41353600
C -8.81524000 -0.62279900 1.88710000
H -8.89331500 0.36162000 1.43022400
H -8.79049900 -0.52988400 2.97921900
C -10.36652100 -2.49855600 2.15661600
O -9.69578400 -3.00972800 3.06071500
C -11.70118500 -3.11540500 1.70952800
N -9.99050700 -1.38393900 1.49035400
H -10.60428000 -0.96959300 0.78950700
C 10.36652100 2.49855600 -2.15661600
C 8.81524000 0.62279900 -1.88710000
H 8.79049900 0.52988400 -2.97921900
H 8.89331500 -0.36162000 -1.43022400
C 7.51389100 1.26903000 -1.39716700
C 5.94307600 3.12349300 -1.70630300
H 6.01144700 4.10697100 -2.17139800
O 9.69578400 3.00972800 -3.06071500
N 9.99050700 1.38393900 -1.49035400
H 10.60428000 0.96959300 -0.78950700
O 6.89341900 0.84492200 -0.41353600
N 7.09643000 2.33297500 -2.11661000
H 7.75609000 2.71017200 -2.79712300
H 5.95032700 3.23222500 -0.61503500
C 8.46512300 -0.65079000 1.86270000
H 8.49134100 -0.55521700 2.95454700
H 8.38796900 0.33263900 1.40356700
C 5.57464500 -3.13948500 1.69398000
H 5.50443400 -4.12189500 2.16105900
N 10.18190000 -2.36282800 2.09383100
H 9.52251900 -2.73743900 2.77604300
C 9.76509100 -1.29982500 1.37261100
O 10.38496700 -0.87853400 0.38733600
N 7.28807000 -1.41096200 1.46920400
H 6.67522900 -0.99806000 0.76672300
O 7.57956800 -3.03157100 3.04549700
C 6.90999300 -2.52255500 2.13928600
C 4.60837500 2.50486300 -2.15096000
O 3.93673000 3.01351600 -3.05583200
N 4.23316000 1.39196300 -1.48134800
C 3.05786600 0.62929600 -1.87515200
H 4.84747000 0.97971400 -0.77974100
H 3.03230100 0.53336000 -2.96701300
H 3.13655200 -0.35387000 -1.41561100
C 1.75663400 1.27635000 -1.38604900
O 1.13769500 0.85505000 -0.40008500
N 1.33816500 2.33795100 -2.10857900
H 1.99736500 2.71333900 -2.79060200
H 5.56844900 -3.25062600 0.60295000
N 4.42163900 -2.34692300 2.10155800
H 3.76228800 -2.72130400 2.78398700
C 4.00360800 -1.28577400 1.37811200
O 4.62252100 -0.86580700 0.39153300
C 2.70281400 -0.63749200 1.86690700
H 2.62478800 0.34536000 1.40658600
H 2.72857000 -0.54064600 2.95868500
C 1.15018600 -2.51111000 2.14474800
O 1.82078500 -3.01900600 3.05084500
N 1.52680500 -1.39948800 1.47383900
H 0.91316500 -0.98761900 0.77142900
H 11.26328400 -4.13885700 2.15030800
H 11.32626800 -3.26579400 0.59313600
H 11.70769000 3.22217600 -0.61806800
H 11.77061600 4.09968800 -2.17276600
N -12.85423500 -2.32488000 2.12048600
H -13.51343800 -2.70201700 2.80151400
H -11.77061600 -4.09968800 2.17276600
H -11.70769000 -3.22217600 0.61806800
H -11.32626800 3.26579400 -0.59313600
H -11.26328400 4.13885700 -2.15030800
C 34.43429900 -2.88343400 2.09786000
C 34.70995400 2.52670400 -1.78152500
C -34.70995400 -2.52670400 1.78152500
C -34.43429900 2.88343400 -2.09786000
H -34.28952100 3.79034600 -1.49788300
H -34.91045300 3.16226100 -3.04405500
H -35.08575200 2.19718200 -1.55507200
H 34.91045300 -3.16226100 3.04405500
H 34.28952100 -3.79034600 1.49788300
H 35.08575200 -2.19718200 1.55507200
H 35.42206200 2.34424700 -2.59269000
H 34.57067300 3.60984000 -1.70777400
H 35.12741600 2.14960000 -0.84352500
H -34.57067300 -3.60984000 1.70777400
H -35.42206200 -2.34424700 2.59269000

```
H -35.12741600 -2.14960000 0.84352500
```

References

1. McGuire, R.F.; Momany, F.A.; Scheraga, H.A. Energy Parameters in Polypeptides. V. Empirical Hydrogen Bond Potential Function Based on Molecular Orbital Calculations. *J. Phys. Chem*. **1972**, *76*, 375–393.
2. Herzberg, G. *Molecular Spectra and Molecular Structure. I. Spectra of Diatomic Molecules*; Van Nostrand: Princeton, NJ, USA, 1950.
